# Lay conceptions of “being moved” (“bewegt sein”) include a joyful and a sad type: Implications for theory and research

**DOI:** 10.1371/journal.pone.0276808

**Published:** 2022-10-27

**Authors:** Ines Schindler, Valentin Wagner, Thomas Jacobsen, Winfried Menninghaus

**Affiliations:** 1 Department of Language and Literature, Max Planck Institute for Empirical Aesthetics, Frankfurt am Main, Germany; 2 Experimental Psychology Unit, Helmut Schmidt University/University of the Federal Armed Forces Hamburg, Hamburg, Germany; University of Bergen, NORWAY

## Abstract

Being moved has received increased attention in emotion psychology as a social emotion that fosters bonds between individuals and within communities. This increased attention, however, has also sparked debates about whether the term “being moved” refers to a single distinct profile of emotion components or rather to a range of different emotion profiles. We addressed this question by investigating lay conceptions of the emotion components (i.e., elicitors, cognitive appraisals, subjective feelings, bodily symptoms, and consequences for thought/action) of “bewegt sein” (the German term for “being moved”). Participants (*N* = 106) provided written descriptions of both a moving personal experience and their conceptual prototype of “being moved,” which were subjected to content analysis to obtain quantitative data for statistical analyses. Based on latent class analyses, we identified two classes for both the personal experiences (joyfully-moved and sadly-moved classes) and the being-moved prototype (basic-description and extended-description classes). Being joyfully moved occurred when social values and positive relationship experiences were salient. Being sadly moved was elicited by predominantly negative relationship experiences and negatively salient social values. For both classes, the most frequently reported consequences for thought/action were continued cognitive engagement, finding meaning, and increased valuation of and striving for connectedness/prosociality. Basic descriptions of the prototype included “being moved” by positive or negative events as instances of the same emotion, with participants in the extended-description class also reporting joy and sadness as associated emotions. Based on our findings and additional theoretical considerations, we propose that the term “being moved” designates an emotion with an overall positive valence that typically includes blends of positively and negatively valenced emotion components, in which especially the weight of the negative components varies. The emotion’s unifying core is that it involves feeling the importance of individuals, social entities, and abstract social values as sources of meaning in one’s life.

## Introduction

The social functional approach to emotions is based on the assumption that emotions enable the formation, maintenance, and negotiation of relationships and attachments [1, 2]. Emotions play an important role in realizing and balancing two competing social goals: social connectedness and cooperation (affiliation function) versus autonomy and competition (distancing function) [3].

### Being moved as a social emotion

Theorizations and research on the emotional state of being moved [4–12] as well as a more recently proposed social emotion called “kama muta” (a Sanskrit phrase meaning “moved by love”), which represents a largely overlapping construct [12–18], have revealed that being moved should be added to the list of social emotions with an affiliation function. Being moved helps orient and transform relationships, and these relationships extend beyond close interpersonal relationships to attachments to other humans and animals, to larger communities, nations, and all of humanity, to values that are shared within a community, and to entities (e.g., the earth, nature).

Researchers who have aimed to establish being moved as a well-defined psychological construct converge on the assumption that this term designates a specific emotional response and not just any strongly felt emotion whatsoever [4–10, 19–23]. However, little agreement has been reached regarding the characteristic features that are distinctive of this emotion [5–7, 9, 12, 15]. Rather, these discussions have raised the question of whether the term “being moved” actually “refers to a single specific emotion, or … can be used to refer to several different emotions that share some common feature” [5 p. 357]. In the present study, we contribute to this discussion by examining laypersons’ concepts of “being moved,” more specifically, laypersons’ concepts associated with the German term for this emotion, “bewegt sein.” We investigate whether our study participants’ descriptions of their own recalled experiences and of their cognitive prototypes for “bewegt sein” point to a highly specific emotional experience or rather to different emotional experiences that share some features. Before turning to our study, we discuss more generally what an emotion is and how it can be determined whether two or more emotion episodes are instances of the same emotion.

### Defining and distinguishing emotions

The definition of the emotion construct continues to be a controversial issue. Different families of psychological emotion theories (which, in addition to the social functional approach mentioned above, include evolutionary/basic emotion, network, appraisal, and constructionist theories) offer different conceptualizations of emotion [see, e.g., 24, 25]. Rather than siding with one theory, we adopt a perspective that builds on the converging assumptions across conceptualizations. According to integrative multi-component accounts [1, 25–29], emotions are overlapping networks of continuously changing emotion components, including cognitive appraisals, physiological responses, motor expressions, action tendencies, and subjective feelings. The subjective feelings integrate and represent (in non-verbal form) changes in the other components and may or may not be categorized or labeled with a specific emotion term. On this view, an emotion term does not designate a “real entity” or “natural kind” that causes the synchronized changes in emotion components. Rather, a label for an emotion might be viewed as a dependent variable [29, 30]: It is assigned based on the resemblance of a specific emotion episode to the configuration of emotion components stored in memory as prototypical for this label. Of necessity, the resulting categorization is fuzzy: As every emotion consists of a component network that partially overlaps with the component networks of other emotions, it is not possible to fully and neatly demarcate one emotion from all others.

Based on this account of emotions, two emotion episodes might be considered instances of the same emotion and be labelled with the same emotion term if their feature profiles across the different emotion components are largely similar. While this may sound like a reasonable rule, laypersons do not consistently follow it. There are cases in which emotion episodes with largely overlapping component feature profiles are labeled differently and cases in which emotion episodes with limited overlap in their component feature profiles are labeled with the same emotion term [31, 32]. On the one hand, this may happen because the experiencer has incomplete insight into all of the emotion components, for instance, bodily and expressive changes that are not consciously perceived. On the other hand, given that the main function of emotions is to maintain or change relations between an experiencing subject and the objects, other persons/groups, and events in a specific situation [3, 33], it is crucial to make sense of, categorize, and label an emotion’s core appraisal and function. In this case, labeling an emotional experience might serve to highlight different interpretations of a highly similar non-verbal subjective feeling, or it might even serve to highlight a similar function of rather dissimilar subjective experiences.

Against this background, our study pursued the following question: Do subjective reports of experiences that are intuitively termed “bewegt sein” feature one largely similar emotion component profile or rather a set of different profiles?

### Does “being moved” have one emotion component profile or several?

The study of lay conceptions of emotions has already proven fruitful, for instance, in research on nostalgia [34], the sublime [35], gratitude [36], and various positive emotions, including tenderness, empathy, and different forms of love [37]. As lay concepts of emotions are invariably tied to vernacular verbal expressions, they should not be confused with rigid scientific constructs [38]. Throughout this manuscript, we highlight this distinction by placing the vernacular expression “being moved” between quotes and referring to the scientific construct of being moved without quotes. While this distinction is important, we also need to consider that verbal labels are part of how emotional experiences are communicated and reflected upon and are central to self-report measures of emotion. Moreover, applying an emotion label to an experience may contribute to and even alter the emotional experience [39–41]. It is therefore worth considering the characteristics of emotional states for which laypersons find the label “being moved” most fitting.

We addressed our study’s question by asking participants to freely describe a specific emotionally moving experience and to characterize their general concept of “being moved.” We conducted a content analysis [42] to derive quantitative data from participants’ open responses. Subsequently, we ran latent class analyses (LCAs; see, e.g., [43, 44]) to address our central research question: Did all participants characterize “being moved” in a similar way, or did different subgroups (i.e., latent classes) of the participants offer different characterizations?

Theoretical approaches and empirical findings on being moved (and kama muta) agree on the emotion’s prototypical bodily expressions and sensations as well as on action tendencies and functions. People’s eyes become moist and they may shed tears, they feel choked up and get a lump in their throat, they experience goosebumps and chills, and they have a warm feeling in their chest or throughout their body [4, 5, 9, 11, 17, 23, 45–49]. Being moved serves to build up and strengthen commitment and devotion to relationships and extended attachments and thereby helps tie a community together [4, 5, 9, 15, 23]. All approaches further agree that being moved is an emotion that people like to experience, seek to re-experience, and seek to share with others [4, 5, 9, 10, 15, 19, 45, 47, 49, 50]. Despite this considerable agreement, two emotion components of being moved remain the subject of debate: (1) elicitors and associated appraisals, and (2) the valence, as evident in the subjective feeling of being moved.

#### What exactly elicits states of being moved?

The emotional state of being moved is notable for its broad range of elicitors. These comprise significant relationship and life events (such as births, deaths, marriages, separations, farewells, and reunions), natural catastrophes and human misery, overcoming hardship and achievement against the odds, moral virtue and courage, and stimuli from the domain of aesthetics, such as art, music, film, digital games, and nature [4, 8, 9, 17, 20, 49, 51–54]. At first glance, this diversity of eliciting events makes it difficult to identify the cognitive appraisals that occur in most cases. It might seem even more difficult to specify a “core relational theme,” defined by Lazarus [55] as an integration of all individual appraisal components into a summary of the emotion’s central meaning regarding the relationship between the person and their environment. Nevertheless, some proposals have been made for the core relational theme of being moved (see [6, 12] for reviews).

According to Cova and Deonna ([4, 5]; see also [21]), people feel moved when a positive core value stands out in the circumstances that triggered the emotion as well as when they appraise a behavior as surpassing a value-related internal standard [45]. Deonna recently provided a slightly modified variant of this definition: “[W]e are moved when we are struck by the positivity or goodness of a specific core positive value’s presence” [56 p. 62].

Menninghaus and colleagues [8, 9, 50, 57] suggested that being moved is specifically associated with social concerns and (pro-)social values rather than any core value, and hence might primarily be considered an “attachment emotion” [9 p. 8]. Similarly, it has been proposed that kama muta is evoked by the perception of a sudden intensification of a communal sharing relationship in which people feel closely related and distribute resources according to need [13, 15, 17, 18, 47, 58, 59].

These proposals have led to an ongoing debate: Is being moved specifically related to (pro)social and attachment-related elicitors, which can, but do not have to, exemplify core values? Or is a core value necessary to elicit feelings of being moved, with every core value having the capacity to move people? Studies by Strick and van Soolingen [21] and Landmann et al. [45] demonstrated that relationships and manifestations of love moved people more intensely than outstanding achievements, willpower, and beauty. However, the latter elicitors were also found to have the capacity to move people.

In an attempt to integrate these positions, Cullhed has proposed “dearness” as the core relational theme of being moved: “We are moved by something when we perceive its dearness, or when we perceive the dearness of an idea that it manifests” [6 p. 115]. According to him, being moved is an attachment emotion that is elicited not only by a person’s interpersonal (basic) attachments, but also by his or her extended attachments. Such extended attachments include relationships to abstract ideas and cultural objects [60], which we “love in the same way we love a child, parent, sibling, or friend” [6 p. 116]. While Cullhed has offered a theoretical proposal that might allow reconciling the core value and attachment accounts of being moved, it is not yet clear to what extent this proposal will be adopted by other scholars and thus lead to greater convergence in future work on being moved.

#### How does it feel to be moved?

Accounts of being moved further disagree on the subjective feeling of being moved, in particular, on the involvement of negative affect in being moved. Is being moved a purely positive emotion––in which case co-occurrent negatively valenced feelings are not an integral part of the emotion––or can it actually include negatively valenced emotion components without this compromising its overall positive valence?

Because the co-occurrence of positive and negative emotional ingredients is often discussed under the term “mixed emotion,” we first discuss how this term is conceptualized in the literature on being moved. Some publications report that emotions experienced in everyday life are often mixed [cf. 30]. This conclusion is based on the observation that the specific configuration of emotion components during one emotion episode often matches the semantic profile of more than one emotion term and is therefore labeled with two or more terms. The terms that are chosen typically designate emotions of the same valence (such as combinations of joy and pride or of anger and disgust). In contrast to such a broad conceptualization of mixed emotions, a narrower definition limits mixed emotions to emotion episodes that include emotional ingredients of opposite valence [61–63]. Such episodes have well been reported, if only for a minority of everyday emotional experiences [30, 64].

In principle, the co-presence of positive and negative affect in the same emotion episode can be modeled as an alternation or as a strictly simultaneous co-activation [61, 62]. The alternation account proposes that, while positive and negative affect are not felt at the very same moment, they are still experienced in such rapid succession that the subjective percept is that of a joint experience of the two. The co-activation account recognizes that different emotion components might have different valences and that, for that reason, the positive and negative valences of different appraisals and action tendencies can be experienced simultaneously [63, 65]. For being moved, the resulting questions are (1) whether states of “being moved” include moments of negative valence or emotion components with negative valence and (2) whether these negatively valenced moments and components are an integral part of being moved or should rather be conceptualized as belonging to a different emotion.

Kama muta is defined as a purely positive emotion elicited by increases in felt closeness [13, 15–18, 47, 58]. While some eliciting events might be predominantly negative, kama muta is limited to positive moments. A contrasting view holds that there are predominantly joyful and predominantly sad variants of being moved, and that, in the trajectory of such episodes, positive and negative feelings can either co-occur or alternate [8–11, 19, 46, 66, 67]. Importantly, inclusion of a negative emotional component is not viewed as rendering an episode of being moved ambivalent. Rather, the overall valence remains positive [9, 66].

There is empirical evidence that both positive and negative affect as well as both joy and sadness are reported during moving experiences [9, 11, 16, 19, 47, 68, 69]. Moreover, negative affect can not only occur as a part of being moved but can also contribute to its overall pleasurability. Specifically, for episodes of being profoundly (and pleasurably) moved by poems and film-clips, studies have reported a co-occurrence of chills, tears, increased activity of the corrugator facial muscle (indicating negative affect), and activation of the zygomaticus facial muscle (indicating positive affect) and the neural reward circuitry [23, 46, 66]. Kimura et al. [70] found increased corrugator activity in response to moving film clips. Mori and Iwanaga [49] reported single and mixed episodes of chills and tears in response to moving music. In contrast, Zickfeld et al. [48] obtained evidence only for increased zygomaticus activity but not for increased corrugator activity during moments of kama muta. This may, however, be related to the fact that the video stimuli used in Zickfeld et al.’s study exclusively presented joyfully moving scenarios, but not sadly moving ones.

Strick and van Soolingen [21 Study 1] conducted separate assessments of being negatively moved by unfavorable circumstances (such as separation, loss, debilitating disease, poverty, and war) and of being positively moved by the emergence of a positive value in these very circumstances. Cova et al. [5], who agree that people can feel negatively moved or positively moved, consider these responses as two distinct affective phenomena in that being negatively moved feels cold and unpleasant, whereas being positively moved feels warm and pleasant. Similar to the kama muta approach, Cova and Deonna [4, 5, 7, 56] proposed that the scientific construct of being moved should be limited to purely positive and mixed (occasions featuring positive and negative elements) cases. Purely negative cases are instead instances of sadness that are (perhaps erroneously) labeled as “being moved” because of overlapping emotion components (in particular, tears and a lump in the throat).

### The present study: Emotion components of being moved

To the best of our knowledge, we are the first to conduct a study collecting laypersons’ descriptions of all emotion components of “bewegt sein” in an open response format. Participants freely reported on the cognitive appraisals, subjective feelings, bodily symptoms, and consequences for thought and action that they regarded as characteristic of “being moved.” We analyzed the data via content analysis [42], that is, we developed a coding scheme to quantify the qualitative information included in the open descriptions [71]. While Cova and Deonna [4, 5] also collected open descriptions of “being moved,” their study was based on the English term, included much shorter answers that did not address the full range of emotion components, and did not include an in-depth content analysis. Based on our coded data, we addressed our central research question through LCA: Are there latent classes of “being moved” with different emotion component feature profiles? This empirical question is linked to an important theoretical issue: Should the evidence point to more than one latent class, what would this imply for the scientific construct of being moved and future research on this construct?

To address the question of whether social elicitors are central to being moved, we considered two fundamental dimensions underlying models of social emotions, social cognition, needs, motives, values, traits, and behavior. These dimensions have previously been designated by a variety of conceptual pairs such as autonomy/competition and social connectedness/cooperation, agency and communion, autonomy and relatedness, competence and sociability/morality, and competence and warmth [3, 72–76]. Overall, the two dimensions represent (1) characteristics linked to individuals striving to master the environment, including competence, achievement, and self-direction, and (2) characteristics linked to striving for connectedness and cooperation with others, including affiliation, benevolence, and prosociality. Accordingly, we coded our participants’ responses as reflections of agency (including values such as courage, willpower, achievement, and health, which have been listed as elicitors of being moved [4, 21, 45]) and connectedness/prosociality (including closeness and communal sharing, which are central to kama muta [13, 15, 18, 47, 58]).

To evaluate the valence of eliciting conditions, we analyzed the participants’ event descriptions with regard to positive salience (emerging values, fulfilled needs) or negative salience (incongruence with or violation of values, unfulfilled needs) of agency and connectedness/prosociality. It is important to note that in this context, the “negative salience” codes do not imply that agency and connectedness/prosociality are themselves negatively valenced. Rather, negative events can call to mind the importance of positive values and evoke a desire to conform with and defend these values.

We further coded participants’ self-reported subjective feelings using various categories, including basic affective dimensions (pleasant/unpleasant valence; low/high arousal) and discrete emotions such as joy, sadness, and relatedness/empathy/appreciation. Moreover, we were interested in whether participants would spontaneously characterize “being moved” as a “mixed” or “complex” emotion and included a code to reflect this. English translations of all code categories included in the German coding manual together with exemplary answers and frequencies for each code are presented in S1 Table.

## Methods

The study was conducted online using SoSci Survey (https://www.soscisurvey.de/). The German questionnaire along with an English translation, the German coding manual, the coded data, and the analysis scripts are available in the Open Science Framework (OSF) at http://doi.org/10.17605/OSF.IO/M8F7D. Our participants’ original written responses are not publicly available. They contain information that could compromise the participants’ privacy and anonymity, and sharing this information would violate the agreement to which the participants consented. The original data can be accessed by researchers upon reasonable request (see Acknowledgments for contact information). Following the ethics statement, we report how we determined our sample size, all data exclusions (if any), all manipulations (if any), and all measures in the study.

### Ethics statement

The study was conducted at Freie Universität Berlin in full accordance with the World Medical Association’s Declaration of Helsinki and the Ethical Guidelines of the German Association of Psychologists (DGPs). Formal ethical approval for the type of research reported in this paper is not required by these guidelines, by German law, or by the Freie Universität Berlin (as confirmed in writing by the local ethics committee of the Department of Education and Psychology). The authors evaluated this study as not creating any harm to the participants. Under this assumption—which, according to German law, is at the full discretion of the authors and for which they hence assume full responsibility—and in line with the above-mentioned rules and customary procedures, a formal ethics approval or waiver of such an approval was not required and hence not requested by us.

The participants were explicitly informed about the task they were expected to perform, the anonymity of the data obtained through this task, the fully voluntary nature of their participation, and their right to withdraw from the study at any time. Thereafter, they gave their informed consent by clicking the response “yes, I agree.” Only those who consented were allowed to begin the online questionnaire; everyone else was taken to the last page of the questionnaire.

### Participants and procedure

We intended to include about 100 participants to obtain a broad range of emotionally moving experiences while simultaneously keeping the amount of data manageable for coding. Moreover, sample sizes of less than 100 often are not feasible when using LCA [cf. 77]. It should be noted, however, that there is no single value for the minimum required sample size that would fit all LCA models. Depending on characteristics such as the number of classes and the number of indicators in the LCA, the class proportions, and the separation between classes, sample sizes of less than 50 may yield sufficient power, but more than 1,000 participants may also be required to achieve adequate power [43, 78, 79].

The sample was recruited via a mailing list (people interested in participating in studies and participants in earlier unrelated studies) maintained by the research cluster “Languages of Emotion” at Freie Universität Berlin. Data were collected between January 2013 and March 2014. Of the 154 people who consented to participate, 44 did not answer the questions on “being moved.” We also decided not to code the responses of one participant who, for the most part, did not answer our questions but rather commented on something else (such as what we should be doing research on). Three participants were excluded from the analyses because their reported personal experiences suggested that they did not understand “bewegt sein” as referring to a specific emotion. One reported on moving physically (jogging) and two on losing self-control in a conflict situation. Neither of the latter two offered a description of the “being-moved” prototype (one explicitly stated that she could not answer this and that our questions were confusing), suggesting a vague concept of “being moved” as becoming emotional. This resulted in a final sample of *N* = 106 participants for coding and analyses.

The participants were between 18 and 75 years of age (*M* = 34.0, *SD* = 13.3), and 97.2% were native German speakers. The sample predominantly included women (72.6%) and highly educated individuals (91.5% were qualified for college entrance, and 50.0% had graduated from college/university).

In the questionnaire, we first collected demographic data, then asked respondents to recall and describe a moment when they felt moved, and finally asked questions designed to explore their prototypical understanding (mental concept) of “being moved” (henceforth called the “being-moved” prototype). At the end of the study, participants indicated whether they wanted to participate in a lottery drawing for prizes of 20 Euro to be paid via bank transfer. The total payment to participants was 20 times 20 Euro.

Participants took between 5 and 51 minutes, *M* = 23.3, *SD* = 10.2, to complete the questionnaire. They answered seven questions regarding their moment of “being moved” (all questions on “being moved” are included in S1 Appendix): 1) When did they experience it? 2) Where were they, and what was the situation? 3) What happened at that moment? 4) What caused them to feel moved? 5) How did it feel to be moved? 6) What bodily sensations accompanied the feeling of “being moved”? 7) How did their experience of “being moved” influence their subsequent thoughts and actions?

These questions enabled us to collect information for coding eight categories characterizing experiences of being moved. Question 1) was for category 1 “time.” The combined responses to questions 2) and 3) served as a basis for coding category 2 “eliciting event or scenario,” and category 3 “respondent’s role in the event” (i.e., as participant or witness). Questions 4), 5), and, respectively, 6) provided the basis for coding category 4 “cognitive appraisals,” category 5 “subjective feelings,” and, respectively, category 6 “bodily symptoms.” Responses to question 7) were coded in category 7 “cognitive consequences” and category 8 “action tendencies.”

After answering the seven open questions, participants rated how moved they were during the moving moment reported in the study, ranging from 1: *very mildly* to 5: *very strongly*. This rating confirmed that most respondents (92.2%) felt strongly or very strongly moved, *M* = 4.48, *SD* = 0.73. Finally, participants answered two questions regarding their prototypical understanding of “being moved:” 1) What characterizes “being moved”? 2) How would they describe the eliciting situation in general terms (in one sentence)?

### Coding

We employed a combined top-down and bottom-up approach [cf. 80] to develop the German coding manual [71], which is available in the OSF. The (S1 Table) includes English translations of the code labels and of examples of text segments to which the code was assigned as well as the number and percentage of participants to whose written responses we assigned the code at least once.

During the theory-driven, top-down part, we derived codes based on work on being moved that was available to us in 2014. Specifically, the initial codes for eliciting events (category 2) and emotion components (categories 4–8) were based on prototypical elicitors, appraisals, bodily symptoms, and action tendencies of being moved that were reported in the literature [4, 8–10, 20, 22, 33]. In addition, we considered work on related emotions, such as moral elevation [81], poignancy [82], and nostalgia [83], and relevant theories regarding the dimensions of agency and connectedness/prosociality [72] and attachment [84]. We further inspected the GRID instrument [85], which provides items to assess the full range of emotion components for any emotion, in order to include components that were not listed in the literature on being moved but might be of general relevance. To investigate the possibility that “being moved” results from elicitors and appraisals of opposite valence, the codes were developed as pairs. For each code that we identified, we included a second code that reflected its opposite or complement. For instance, elicitors could be coded as “beginning of life” (code 2111) or “end of life” (code 2112), appraisals as “familiar/expected” (code 4111) or “unfamiliar/unexpected” (code 4112), and subjective feelings as “pleasant” (code 51011) or “unpleasant” (code 51012).

For the bottom-up part, the first author inspected all participants’ responses and selected ten participants whose reports together covered a broad range of elicitors and emotion components. These responses were used for identifying additional codes, for discussing and refining codes, and for training the coders. Two independent coders then coded the responses of the remaining 96 participants; these responses were not employed to develop the manual (κ = .74; disagreements were resolved by the first author).

The final coding manual includes a total of 165 different codes in eight major code categories (as described in the previous section). Each code has a number (2–5 digits); the first digit always gives the major category (1–8). The 6 codes in category 1 “time” and 10 codes in category 3 “respondent’s role in the event” could only be assigned when coding descriptions of a moving personal experience but not when coding descriptions of the “being-moved” prototype. The remaining 149 codes could be assigned to both moving personal experiences and characterizations of the prototype. For these codes, the second digit gives the subcategory (1–5; only when there are subcategories), the following digits number the codes within (sub)categories, and the last digit (1 or 2) indicates the pole (e.g., 1 = *positive valence*, 2 = *negative valence*) for paired codes (i.e., pairs have the same code number except for the last digit).

Based on the coded data, we created two binary variables per code in categories 2 and 4–8: (1) a variable indicating whether the code was assigned at least once (0 = *no*, 1 = *yes*) in a participant’s response for the personal experience, and (2) a variable indicating whether the code was assigned at least once (0 = *no*, 1 = *yes*) in a participant’s response for the prototype. As codes in categories 1 and 3 were only assigned to personal experiences, there is only one binary variable per code in these categories.

While the overall coder agreement was good, the coders were less reliable in distinguishing between “connectedness” as feeling close to others and appreciating relationships on the one hand, and “prosociality” as doing something for others and making sacrifices for relationships on the other hand. We therefore decided to combine the codes for “connectedness” and “prosociality” in one code for the analysis (in all relevant categories, i.e., 4 “cognitive appraisals,” 7 “cognitive consequences,” and 8 “action tendencies”). For instance, the original codes “positive salience of connectedness” (code 4421) and “positive salience of prosociality” (code 4431) were combined as “positive salience of connectedness/prosociality” (code 4421_31). The respective binary code variable is 1 when code 4421 and/or code 4431 were assigned at least once in a participant’s response and 0 when neither code was assigned.

We combined a few further codes to obtain more inclusive codes and, thus, fewer variables for our analyses. Specifically, within category 3 “respondent’s role in the event,” we skipped the distinction between personally witnessing what another person was experiencing at the moment and learning about some past or expected future experience of another person (given that the respondent was a witness in both cases). We also decided not to maintain the distinctions between recognizing the value of agency or of connectedness/prosociality (category 7 “cognitive consequences”) and actually striving for agency or connectedness/prosociality (category 8 “action tendencies”). This resulted in the compound codes “recognize value of/strive for agency” (code 741_841) and “recognize value of/strive for connectedness/prosociality” (code 751_61_851_61). All code combinations are listed in S1 Table.

### Data analysis

As a first step, we inspected the descriptive information about the time and nature of the moving events reported by the participants as well as their involvement in the events. In a second step, we used Mplus Versions 7.4 and 8.4 [86] to compute LCAs for the coded data to address our central research question of whether there are different classes of moving personal experiences and of the “being-moved” prototype. The LCAs were conducted on the codes for the emotion components of “being moved” (code categories 4–8). LCAs identify classes based on their emotion component profile, that is, based on the pattern across a number of binary variables indicating the code assignment (no/yes). Particularly when the sample size is small, including more indicators (codes) of higher quality (i.e., codes with a high probability of the code assignment, such as .90, in one class and a low probability of the code assignment, such as .10, in other classes) can compensate for the sample size [77]. It should be noted, however, that the number of indicators examined in simulation studies is typically less than 15 [77–79]. Moreover, codes that are rarely assigned in the entire sample are unlikely to be high-quality indicators. For instance, if only 5% of the participants provide an answer to which a specific code is assigned, this would mean that even if this code were a perfect indicator of class membership, the underlying latent class would comprise only 5% of the sample.

In light of these considerations, we decided to include in the LCA only emotion component codes that were assigned to the responses of at least 25% of the participants (see S1 Table). This resulted in the selection of 16 codes for moving personal experiences and hence a relatively large number of indicators. The selected appraisal codes were “unfamiliar/unexpected” (code 4112; 33.0%), “personal relevance” (code 4121; 26.4%), “positive salience of agency” (code 4411; 30.2%), “negative salience of agency” (code 4412; 41.5%), “positive salience of connectedness/prosociality” (code 4421_31; 69.8%), and “negative salience of connectedness/prosociality” (code 4422_32; 41.5%). For the subjective feelings, we selected “uncontrollability/magnitude of emotion” (code 52022; 30.2%), “joy” (code 53011; 49.1%), “sadness” (code 53022; 34.0%), “relatedness/empathy/appreciation” (code 53031; 50.0%), and “mixed/complex emotion” (code 54012; 38.7%). The bodily feelings included “warmth” (code 6011; 28.8%) and “tears” (code 6031; 54.8%). The consequences for thought and action included “keep thinking” (code 721; 33.3%), “finding meaning” (code 731; 25.7%), and “recognize value of/strive for connectedness/prosociality” (code 751_61_851_61; 39.0%).

Five of the eight codes that were applied to at least 25% of the participants’ prototype responses were also included in the LCA for the personal experiences: appraisals of “unfamiliarity/unexpectedness” and “personal relevance” as well as feelings of “joy,” “sadness,” and “uncontrollability/magnitude of the emotion.” In contrast to the descriptions of personal experiences, the descriptions of the “being-moved” prototype rarely included explicit appraisals of agency and/or connectedness/prosociality. Rather, participants wrote primarily about the valence of potential elicitors. They stated that individuals can be moved by positive or negative events without specifying what makes these events positive or negative. Such responses were coded as “pleasant” (code 51011; 34.0%) or, alternately, “unpleasant” (code 51012; 25.2%) subjective feelings, marking the opposite poles of the valence dimension. In addition, prototypical descriptions offered by 27.2% of the participants were coded as “intuition” (code 53081), that is, “being moved” means thinking and acting emotionally and intuitively rather than rationally. To keep this LCA comparable to that for personal experiences, we only added feelings of pleasantness and unpleasantness and dropped four codes that were assigned to the responses of fewer than 10% of the participants: “positive salience of agency” (5.8%), “negative salience of agency” (7.8%), “negative salience of connectedness/prosociality” (1.0%), and “warmth” (5.8%). This resulted in 14 codes being employed in the LCA for the “being-moved” prototype.

We also tested whether different latent classes would result if we included fewer or more indicators for the LCAs on moving personal experiences and the “being-moved” prototype. Even with a different number of indicators, we obtained highly similar latent classes, as illustrated by the two-class models with more indicators presented in S2 and S3 Appendices. As a more stringent selection criterion of 30% of the responses would have led to the exclusion of the theoretically interesting indicators “warmth” and “finding meaning” in the LCA for personal experiences, we decided to stick to the 25% selection criterion.

We ran LCAs with one to four latent classes. All models were estimated with 5,000 initial stage random sets of starting values and 500 final stage optimizations. We also tried running LCAs with five or more classes, but these ceased to be well identified (a stopping criterion for fitting additional models [44]). For instance, many perturbed starting value runs did not converge, the condition number was very small, and we obtained some very small latent classes (less than 5% of the sample). To decide on the number of classes to be extracted, we considered statistical information criteria (Akaike’s Information Criterion [AIC] and Bayesian Information Criterion [BIC]), entropy, the average latent class probabilities for the most likely latent class membership (*AvePP*), and likelihood-based tests. Entropy is a standardized summary measure of the classification accuracy of placing participants into classes based on their posterior probabilities (range 0–1). Entropy values greater than .800 and *AvePP* values greater than .700 indicate good classification accuracy and well-separated classes [cf. 43, 44]. The *p*-value of a likelihood ratio test (LRT) gives the likelihood that the data would have been generated by a model with one less class. In sum, smaller values for the information criteria and the *p*-values of LRTs and larger values for entropy and *AvePP* indicate better model fit.

After deciding on the number of latent classes to be extracted, we saved the resulting assignments of participants to the latent classes for moving personal experiences and for the “being-moved” prototype in our data file. We ran Mann-Whitney U tests with IBM SPSS Statistics Version 24.0 to test for significant differences in code assignments between the latent classes. Due to the exploratory nature of these analyses and because the sample size required separate tests for each code (rather than including all 16 or 14 codes in a logistic regression analysis), we applied a Bonferroni-corrected alpha level of *p* < .0031 for the moving personal experiences and of *p* < .0036 for the “being-moved” prototype.

## Results

In this section, we first provide an overview of the eliciting events that were reported for moving personal experiences. Then we present the LCAs conducted on the emotion component codes that were most frequently assigned to the reports of moving personal experiences and the tests for significant differences between the classes that were extracted. Finally, we report the results of the LCAs and the tests for class differences in the descriptions of the “being-moved” prototype.

### Moving events

We asked participants to recall emotionally moving situations they had experienced during the previous months. In line with this instruction, 71.7% of the participants reported a moving event that they had experienced in the preceding six months (codes 11–13 in S1 Table). The remaining participants either reported events that happened from between slightly more than six months to several years before (19.8%; codes 14–16) or provided vague or no answers, which did not allow us to assign a time code (8.5%).

**Fig 1:**
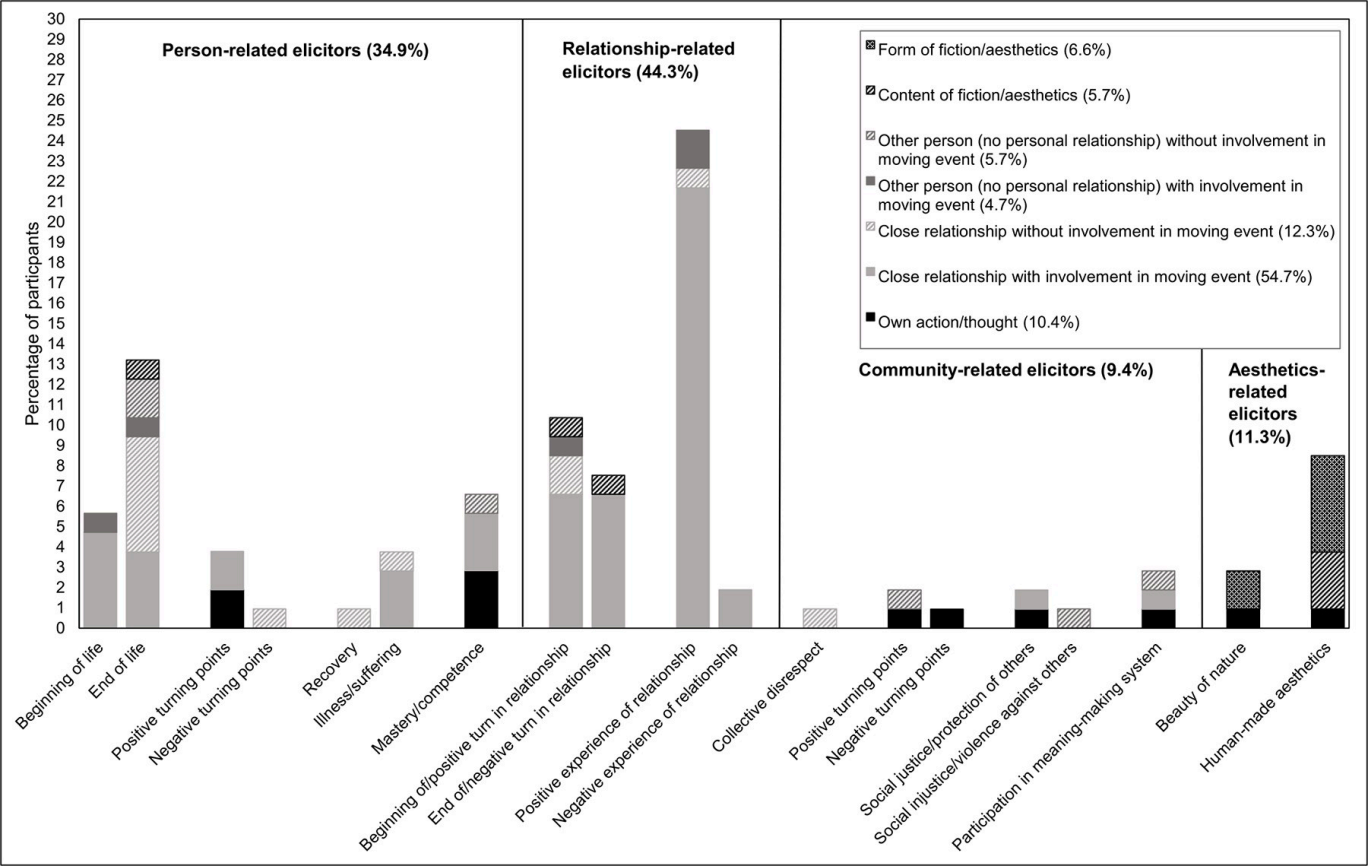
Types of moving events (category 2) broken down by involvement in event (category 3).

We coded the eliciting events (code category 2 in S1 Table) and the respondents’ involvement in these events (as a participant or witness; code category 3 in S1 Table). It should be noted that the codes in categories 2 and, respectively, 3 are mutually exclusive: we assigned only one elicitor and one involvement code for each event. Fig 1 provides an overview of the resulting combinations of elicitors and involvement.

As shown in Fig 1, the most frequent events reported were “positive experience of relationship” (code 2221; 24.5%; e.g., surprise parties, receiving special gifts), “end of life” (code 2112; 13.2%; e.g., funerals, news of someone’s death), “beginning of/positive turn in relationship” (code 2211; 10.4%; e.g., reunions, weddings), “human-made aesthetics” (code 2421; 8.5%, e.g., dance performances, music), “end of/negative turn in relationship” (code 2212; 7.5%; e.g., farewells, break-ups), “mastery/competence” (code 2141; 6.6%; e.g., completing an exam), and “beginning of life” (code 2111; 5.7%; e.g., giving/witnessing birth). Taken together, most of the reported elicitors were experiences in or of relationships (codes 2211–2222; 44.3%).

We further coded for whether participants were involved in the event, that is, they were moved while interacting with another person, or witnesses of the event, that is, they were moved by observing or otherwise learning about an event in which they did not take part. Most participants (69.8%) were personally involved in the moving event they reported. Specifically, 54.7% of the participants were moved by interactions with close others (code 321), 4.7% by interactions with persons to whom they were not personally related (code 331), and 10.4% by their own actions and thoughts (codes 311 and 312). The remaining 30.2% of the participants were in a witness position, either directly observing or otherwise learning about something that (had) happened to a close other (code 322_23; 12.3%) or another person to whom they were not personally related (code 332_33; 5.7%), or during an aesthetic experience of art or nature (codes 341 and 342; 12.3%).

### Latent classes of moving personal experiences

Table 1 reports the fit statistics of the LCA models with one to four latent classes for both moving personal experiences and the “being-moved” prototype. The fit indices did not yield full agreement on the best number of classes. While the AIC and sample-size adjusted BIC decreased with an increasing number of classes, the BIC increased. Following Nylund et al. [87], we placed greater weight on the parametric bootstrapped LRT and BIC than on the other fit statistics. Overall, the two-class models yielded a better fit than the one-class models for both moving personal experiences and the “being-moved” prototype. When moving from one to two classes, we found the greatest drop in the AIC and sample-size adjusted BIC, while a simultaneous increase in the BIC was negligible. The entropy values and *AvePP* indicated well-separated classes for all tested models. The LRTs showed that the data most likely would not have been generated by a one-class model. The LRTs did not provide support for the superiority of a three-class model over a two-class model or of a four-class model over a three-class model.

**Table 1:** Model comparisons for one to four latent classes.

	Number of classes			
Fit index	1	2	3	4
*Moving personal experience (16 codes*, *N = 106)*				
AIC	2199	2156	2148	**2135**
BIC	**2242**	2244	2281	2314
Sample-size adjusted BIC	2191	2139	2123	**2102**
Entropy	–	.863	.860	**.894**
*AvePP*_class1_	1.000	.976	.910	.986
*AvePP*_class2_	–	.926	.960	.962
*AvePP*_class3_	–	–	.921	.923
*AvePP*_class4_	–	–	–	.895
Vuong-Lo-Mendell-Rubin LRT: *p*-value	–	**.053**	.373	.141
Vuong-Lo-Mendell-Rubin adjusted LRT: *p*-value	–	**.055**	.381	.145
Parametric bootstrapped LRT: approximate *p*-value	–	**.000**	.109	.040
*“Being-moved” prototype (14 codes*, *N = 103)*				
AIC	1554	1515	**1494**	**1494**
BIC	**1590**	1591	1610	1650
Sample-size adjusted BIC	1546	1500	1471	**1463**
Entropy	–	**1.000**	.972	.956
*AvePP*_class1_	1.000	1.000	1.000	.984
*AvePP*_class2_	–	1.000	.969	.971
*AvePP*_class3_	–	–	.989	.967
*AvePP*_class4_	–	–	–	.982
Vuong-Lo-Mendell-Rubin LRT: *p*-value	–	**.005**	.687	.313
Vuong-Lo-Mendell-Rubin adjusted LRT: *p*-value	–	**.006**	.691	.320
Parametric bootstrapped LRT: approximate *p*-value	–	**.000**	**.000**	.600

*Note*.–means that this statistic is not applicable to this model. AIC = Akaike’s Information Criterion. BIC = Bayesian Information Criterion. LRT = likelihood ratio test. *AvePP* = average latent class probability for the most likely latent class membership. The smallest values for the information criteria and *p*-values of LRTs and the largest values for entropy are highlighted in bold.

As all of the considered fit statistics perform worse in small samples [e.g., 87], we cannot rule out that more than two classes might be found for larger samples. In any event, the data show that there are at least two classes both for the moving personal experiences and for the “being-moved” prototype. In the following, we first present the two-class solution for moving personal experiences. For purposes of comparison, we also include findings for the LCA models with the second-best fit as part of the supporting information. Results for the four-class model for personal experiences are reported in S2 Appendix.

**Fig 2:**
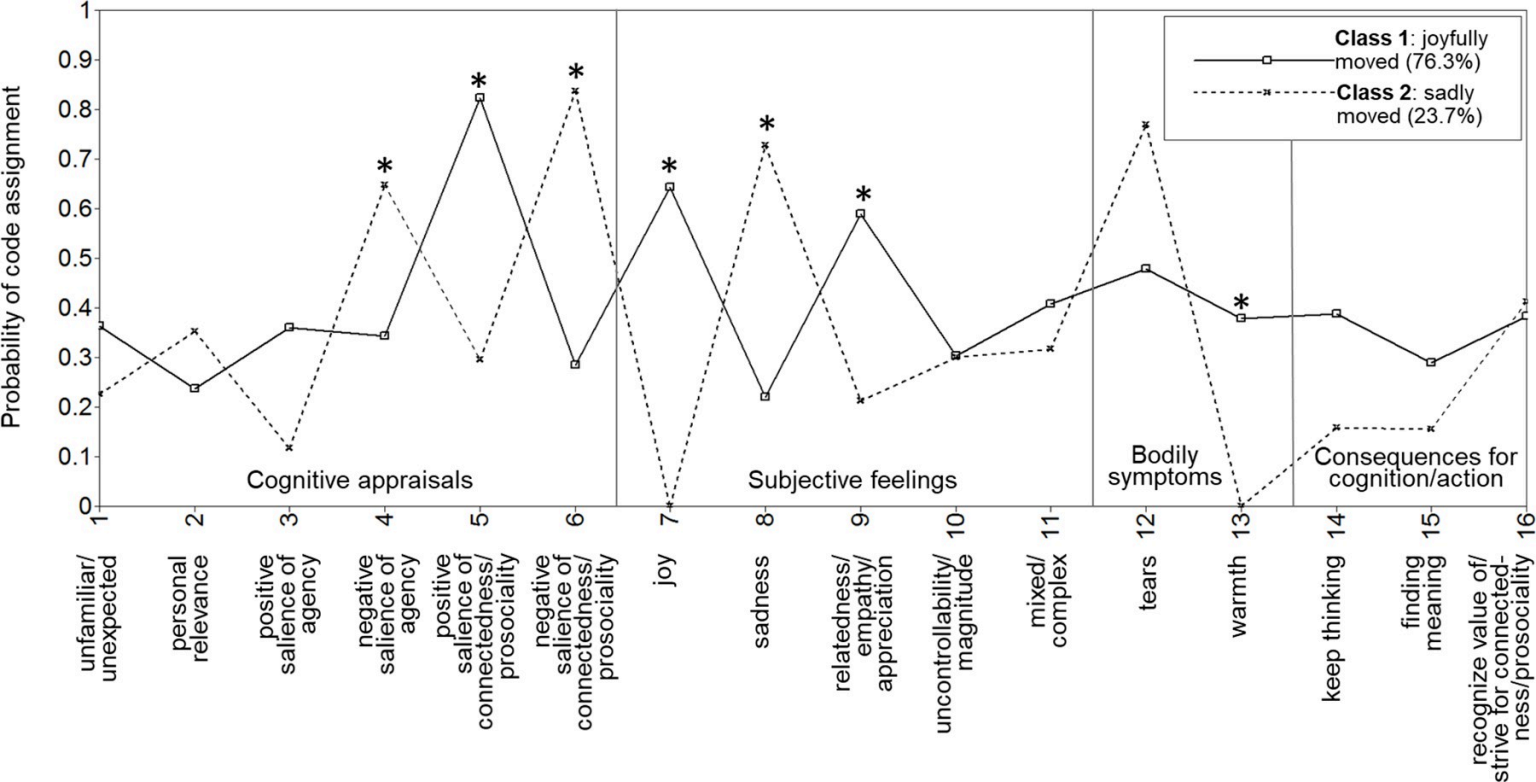
Latent classes for characteristics of “being moved” when describing a personal experience. * Significant difference between class 1 and class 2 at the Bonferroni-corrected alpha level of *p* < .0031.

The two classes found in the LCA for personal experiences can readily be interpreted as corresponding to joyfully moving (class 1) and sadly moving (class 2) experiences (Fig 2). Class 1 shows peaks in the emotion component profile for the appraisal “positive salience of connectedness/prosociality” and the subjective feelings of “joy” and “relatedness/empathy/appreciation.” In contrast, peaks in class 2 are evident for the codes “negative salience of agency,” “negative salience of connectedness/prosociality,” “sadness,” and “tears.” Despite these different profiles, the self-reported intensity of “being moved” was almost identical in the two classes, *M*_class1_ = 4.4 and *M*_class2_ = 4.6 (Table 2).

**Table 2:** Comparisons of latent classes of moving personal experiences and the being-moved prototype.

			Class of personal experience (*N* = 106)						Class of prototype (*N* = 103)					
Variable/Code			1: joyfully moved (*n* = 81)		2: sadly moved (*n* = 25)		Difference class 1 and 2		1: extended description (*n* = 28)		2: basic description (*n* = 75)		Difference class 1 and 2	
Number	Label	Description	*n*/*M*^*a*^	%/*SD*^*a*^	*n*/*M*^*a*^	%/*SD*^*a*^	Test statistic^*b*^	*p* ^*b*^	*n*/*M*^*a*^	%/*SD*^*a*^	*n*/*M*^*a*^	%/*SD*^*a*^	Test statistic^*b*^	*p* ^*b*^
-	gender	female gender	59	72.8	18	72.0	0.01	.934	21	75.0	55	73.3	0.03	.864
-	age	age of participant	33.4	12.2	35.5	16.3	0.46	.502	35.7	15.5	33.3	12.4	0.68	.413
-	education	highest level of education	5.2	0.9	5.4	0.8	907.00	.387	5.3	0.9	5.3	0.9	1033.50	.893
-	moved rating	rated intensity of feeling moved (1–5)	4.4	0.7	4.6	0.8	1.81	.181	4.6	0.5	4.4	0.8	2.19	.142
-	no. codes	number of different codes obtained in categories 4 to 8	13.1	5.1	11.7	2.4	1.81	.182	**9.5**	**4.2**	**5.6**	**2.8**	**29.80**	**< .001**
Category 1: Time of event^c^	recent event	event happened within the last six months^d^	59	80.8	17	70.8	1.06	.303	20	76.9	55	79.7	0.09	.766
Category 2: Eliciting event^c^	positive event	positive eliciting event^e^	**71**	**87.7**	**3**	**12.0**	**51.88**	**< .001**	18	64.3	53	70.7	0.39	.534
Category 3: Respondent’s role in event^c^	involvement in event	participant involved in moving event^f^	61	75.3	13	52.0	4.93	.026	18	64.3	54	72.0	0.58	.448
Category 4: Cognitive appraisals														
4112	unfamiliar/ unexpected	situation or event is unfamiliar, is experienced for the first time, or developed suddenly and/or unexpectedly	30	37.0	5	20.0	840.00	.115	8	28.6	21	28.0	1044.00	.954
4121	personal relevance	event reminds one of one’s own experiences or of persons whom one knows, is related to or experienced as part of oneself	19	23.5	9	36.0	885.50	.216	11	39.3	19	25.3	903.50	.168
4411	positive salience of agency	agency as an ideal is realized in the situation; fulfillment of or positive deviations from expectations for mastery, autonomy, independence, competence, self-esteem, or self-confidence	30	37.0	2	8.0	718.50	.006	2	7.1	4	5.3	1031.00	.728
4412	negative salience of agency	agency as an ideal is challenged or threatened in the situation; unfulfilled needs for agency; negative deviations from expectations for mastery, autonomy, independence, competence, self-esteem, or self-confidence	**27**	**33.3**	**17**	**68.0**	**661.50**	**.002**	2	7.1	6	8.0	1041.00	.886
4421_31	positive salience of connectedness/ prosociality	connectedness/prosociality as an ideal is realized in the situation; fulfillment of or positive deviations from expectations for closeness, love, connection, respect, community spirit, altruism, or prosocial behavior	**66**	**81.5**	**8**	**32.0**	**511.50**	**< .001**	7	25.0	6	8.0	871.50	.021
4422_32	negative salience of connectedness/ prosociality	connectedness/prosociality as an ideal is challenged or threatened in the situation; unfulfilled social needs; negative deviations from expecta-tions for closeness, love, connection, respect, community spirit, altruism, or prosocial behavior	**22**	**27.2**	**22**	**88.0**	**396.50**	**< .001**	0	0.0	1	1.3	1036.00	.541
Category 5: Subjective feelings														
51011	pleasant	pleasant, positive, good feelings; feeling well	18	22.2	2	8.0	868.50	.114	10	35.7	25	33.3	1025.00	.821
51012	unpleasant	unpleasant, negative, bad feelings; feeling ill	**2**	**2.5**	**6**	**24.0**	**794.50**	**< .001**	10	35.7	16	21.3	899.00	.137
52022	uncontrol-lability/ magnitude of emotion	feeling is uncontrollable, strong, deep, wide, overwhelming, sudden, intrusive, or cannot be regulated; person’s body is too narrow for the emotion	25	30.9	7	28.0	983.50	.786	14	50.0	39	52.0	1029.00	.857
53011	joy	feelings of joy, happiness, satisfaction, or cheerfulness	**52**	**64.2**	**0**	**0.0**	**362.50**	**< .001**	**23**	**82.1**	**9**	**12.0**	**313.50**	**< .001**
53022	sadness	feeling sad, depressed, downhearted, sorrowful, or down	**17**	**21.0**	**19**	**76.0**	**455.50**	**< .001**	**28**	**100.0**	**0**	**0.0**	**0.00**	**< .001**
53031	relatedness/ empathy/ appreciation	feelings of affection, closeness, tenderness, sympathy, love, empathy, compassion, admiration, or gratitude	**47**	**58.0**	**6**	**24.0**	**668.00**	**.003**	10	35.7	14	18.7	871.00	.070
54012	mixed/ complex	feeling is characterized as mixed, complex, multi-layered, or complicated	33	40.7	8	32.0	924.00	.435	6	21.4	5	6.7	895.00	.032
Category 6: Bodily symptoms														
6011	warmth	bodily sensations of warmth; warmth in the chest; warm heart	**30**	**38.0**	**0**	**0.0**	**612.50**	**< .001**	1	3.6	5	6.7	1017.50	.553
6031	tears	crying or feeling like crying; tears welling up; moist eyes	37	46.8	20	80.0	660.00	.004	10	35.7	14	18.7	871.00	.070
Category 7: Cognitive consequences														
721	keep thinking	moving experience or emotion is vividly or easily recalled; the person keeps thinking about and will not forget the experience	31	38.8	4	16.0	772.50	.036	2	7.1	11	14.7	971.00	.309
731	finding meaning	moving experience made person realize or guess some truth, which is not characterized any further; person gained some insight or discovered what is important or meaningful	23	28.8	4	16.0	872.50	.205	7	25.0	14	18.7	983.50	.480
751_61	recognize value of connectedness/ prosociality	person realized the importance of specific relationships or connectedness in general, of loving and being loved, caring for others, being prosocial, and advocating for collective values and norms	17	21.3	7	28.0	932.50	.485	0	0.0	3	4.0	1008.00	.285
Category 8: Action tendencies														
851_61	strive for connectedness/ prosociality	person wants or strives to begin, maintain, or improve relationships, seek social connection, show appreciation of, care for, and help others, and stand up for social values	18	23.1	6	24.0	966.00	.925	6	21.4	7	9.3	923.00	.102
Code combination 7 and 8														
751_61_ 851_61	recognize value of/ strive for connectedness/ prosociality	see 751_61 and 851_61	30	37.5	11	44.0	935.00	.563	6	21.4	9	12.0	951.00	.230

*Note*. Significant differences between class 1 and class 2 at the Bonferroni-corrected alpha level of *p* < .0031 (personal experience) or *p* < .0036 (prototype) are highlighted in bold.

^a^ *n* and % are reported for categorical data, *M* and *SD* for continuous data. There are some missing responses for “recent event” (8 in the joyfully-moved and 1 in the sadly-moved class; 2 in the extended-description and 6 in the basic-description class). Here, the percentage of valid responses is reported (instead of percentage of total *n* per class).

^b^Test statistics and *p*-values are for different tests: χ^2^(1) for recent event, positive event, involvement in event, and gender, *F*(1, 100) for age, moved rating, and number of codes, Mann-Whitney U for education and code 4112 to code 75161_85161. For the U tests, approximate *p*-values are reported that are corrected for tied values within the classes. We conducted independent tests for all codes, as running a logistic regression with all predictors was not feasible given the present sample size.

^c^As the descriptions of the being-moved prototype did not include a specific event, these categories cannot be coded. We reported the respective data for the moving personal experience that these participants had described in the first part of the study. We did so in order to show that assignment to the prototype classes was unrelated to personal experiences.

^d^Recent events are defined as all events that happened within the last week, last month, or last six months (codes 11 to 13; see S1 Table); all events happening more than six months ago (codes 14 to 16) were coded as “not recent.”

^e^Positive events are defined as all event codes ending in “1”, negative events have codes ending in “2” (see S1 Table).

^f^Respondent’s role in event codes (see S1 Table) indicating involvement in the moving event are 311, 312, 321, and 331. Codes 322_23, 332_33, 341, and 342 indicate being in a witness position.

#### The joyfully-moved class

As would be expected based on the emotion component profile of class 1, the 81 participants in the joyfully-moved class reported predominantly positive elicitors (87.7%; Table 2) and events in which they were involved (75.3%). The elicitor codes assigned in class 1 were (in descending frequency) “positive experience of relationship” (code 2221; 29.6%), “beginning of/positive turn in relationship” (code 2211; 13.6%), “human-made aesthetics” (code 2421; 9.9%), “mastery/competence” (code 2141; 8.6%), “beginning of life” (code 2111; 7.4%), “end of life” (code 2112; 6.2%), “positive turning points [person]” (code 2121; 4.9%), “participation in meaning-making system” (code 2341; 3.7%), “beauty of nature” (code 2411; 3.7%), “illness/suffering” (code 2132; 2.5%), “positive turning points [community]” (code 2321; 2.5%), “social justice/protection of others” (code 2331; 2.5%), “negative turning points [person]” (code 2122; 1.2%), “recovery” (code 2131; 1.2%), “end of/negative turn in relationship” (code 2212; 1.2%), and “negative turning points [community]” (code 2322; 1.2%).

As can be seen from this list, ten participants (12.3%) in class 1 reported negative eliciting events, five of which were assigned the code “end of life.” When analyzing these reports, a mixed emotional nature of the underlying experiences came to the fore. Some of the events described were about being with a person or pet during their final moments. For instance, one participant (ID 237) reported his wish and his successful effort to see his terminally ill father alive for a last time. While the participant had no longer expected his father to be responsive, his father actually got out of bed for a final embrace. In the words of the participant, he was moved because “the incredible strength my father had to muster to hug me one last time made me realize how important I was to him.”

Another participant (ID 287) reported on attending the funeral of her friends’ friend, who had died at a young age. As she had never met the deceased person herself, she “could view the event from an outside perspective.” An example in the “illness/suffering” category was reported by ID 295, who discovered that her boyfriend had made preparations to commit suicide, but in the end changed his mind.

#### The sadly-moved class

The 25 participants in the sadly-moved class rarely reported positive elicitors (12.0%). Compared to the participants in the joyfully-moved class, they were also less frequently personally involved in the event (52.0%). The specific elicitor codes in this class were “end of life” (code 2112; 36.0%), “end of/negative turn in relationship” (code 2212; 28.0%), “illness/suffering” (code 2132; 8.0%), “positive experience of relationship” (code 2221; 8.0%), “negative experience of relationship” (code 2222; 8.0%), “collective disrespect” (code 2312; 4.0%), “social injustice/violence against others” (code 2332; 4.0%), and “human-made aesthetics” (code 2421; 4.0%).

Closer inspection of the responses given by the three participants in the sadly-moved class who reported a positive eliciting event confirmed that these responses were not misclassified. An intern in a forensic clinic (ID 204) felt moved when, as she left work, a mentally ill offender placed under hospital supervision wished her a nice evening. In her own words, she felt “deeply saddened, touched” by the thought “I am allowed to go home and you cannot.” Another participant (ID 313) felt moved when confessing to her boyfriend that she had been in love with another man and her boyfriend, instead of breaking up, reaffirmed his love for her. The third participant (ID 136) reported that watching the fireworks on New Year’s Eve made her realize that an exhausting year lay behind her. Parallel to negative situations that were reported in the joyfully-moved class, these examples show that blends of positive situations and negative appraisals and feelings occurred in the sadly-moved class.

#### Differences in code assignment between the joyfully-moved and the sadly-moved class

The test statistics and *p*-levels for comparisons of code frequencies between the two classes are reported in Table 2. For the differences that are reported in the text, we also include the Glass rank biserial coefficient (*r*_*Grb*_) along with its 95% confidence interval as one appropriate effect size statistic to use with the Mann-Whitney U test [88, 89]. We employed the R package *rcompanion* [90] to calculate *r*_*Grb*_, which can range between −1 and 1. Values are positive when a code was more frequently assigned in class 1 than in class 2 and negative when a code was more frequently assigned in class 2 than in class 1. Values for |*r*_*Grb*_| in the range .11−.27 are interpreted as small, in the range .28−.42 as medium, and equal to or larger than .43 as large effect [89].

Codes indicating appraised “positive salience of connectedness/prosociality,” *r*_*Grb*_ = .50, 95% CI [.27, .68], feelings of “joy,” *r*_*Grb*_ = .64, 95% CI [.53, .74], and “relatedness/empathy/appreciation,” *r*_*Grb*_ = .34, 95% CI [.11, .52], as well as a bodily feeling of “warmth,” *r*_*Grb*_ = .38, 95% CI [.28, .49], were more frequent in the joyfully-moved class than in the sadly-moved class. Codes indicating appraised “negative salience of agency,” *r*_*Grb*_ = −.35, 95% CI [−.54, −.11], “negative salience of connectedness/prosociality,” *r*_*Grb*_ = −.61, 95% CI [−.74, −.41], and feelings of “sadness,” *r*_*Grb*_ = −.55, 95% CI [−.72, −.33], were more frequent in the sadly-moved class (Fig 2, Table 2). Differences for “positive salience of agency,” *r*_*Grb*_ = .29, 95% CI [.11, .42], “tears,” *r*_*Grb*_ = −.33, 95% CI [−.50, −.11], and the cognitive consequence to “keep thinking,” *r*_*Grb*_ = .23, 95% CI [.02, .39], failed to reach significance at the corrected alpha level, illustrating that our analyses were limited to detecting medium-sized to large effects. The confidence intervals further suggest that these latter effects might be anything from small (or nonexistent) to large.

Regardless of the reported differences in appraisals and subjective feelings, the joyfully-moved and sadly-moved classes showed no significant differences in their reported consequences for thought and action (with the potential exception of “keep thinking”). The most frequently assigned code combination was “recognize value of/strive for connectedness/prosociality.” Additionally, participants reported that they “found meaning” in the experience. The “finding meaning” code was applied when participants stated that “being moved” made them recognize what is true or important, without further specifying the nature of this insight. As expressed by one participant (ID 220): “It’s not so much something that would concretely change my actions, it’s more like a truth capsule that I can go into sometimes to reflect on what really matters.”

#### Co-occurrence of positive and negative elements in moving personal experiences

In addition to analyzing the probabilities of code assignment for the individual codes, we were interested in the co-occurrence or alternation of positive and negative appraisals and subjective feelings during the reported emotional episode, and hence in emotionally mixed experiences (which are, however, typically not ambivalent; see our Introduction). From the 16 codes included in the LCA, we selected “positive salience of agency,” “positive salience of connectedness/prosociality,” and “joy” as indicators of positive experiences and “negative salience of agency,” “negative salience of connectedness/prosociality,” and “sadness” as indicators of negative experiences. We classified an experience as mixed when instances of these two indicators were present in the same report or when a positive or negative experience was also assigned the code “mixed/complex” subjective feeling. While joy and sadness were the predominant emotions in the joyfully-moved and sadly-moved classes, respectively, being moved was a somewhat mixed experience for most participants.

In the joyfully-moved class, 52 participants’ responses (64.2% of the participants in class 1) were classified as mixed. These include reports that were assigned both positive and negative salience of connectedness/prosociality and/or agency. For instance, one participant (ID 244) recalled how her pregnant daughter almost died and had to undergo an emergency caesarian and additional surgeries in order for both baby and mother to be saved. Another participant (ID 160) reported how she at first felt annoyed by an elderly, frail man who needed her help in the university cafeteria, only to find out that this man had been a professor at the same university and had an impressive life to look back on.

We assigned only codes in positive categories to 26 participants’ responses (32.1% of the participants in class 1). For the remaining participants (3.7%), all appraisals and feelings used in the LCA and employed by us to indicate mixed experiences were coded as zero. Purely positive examples included a participant (ID 315) who felt moved by his three-year old nephew who had painted a picture for him “because he loves me so much.” Another participant (ID 304) was moved by watching John Lewis & Partners’ 2013 Christmas advertisement “The Bear and the Hare” on YouTube.

Within the sadly-moved class, 15 participants (60.0% of the participants in class 2) reported positive appraisals and/or subjective feelings that were “mixed/complex” in addition to negative appraisals and sadness (none reported joy). Examples of such mixed cases include a participant who was about to be away for a significant amount of time and said goodbye to her family and friends at the airport (ID 297). Another participant had watched a documentary about the Korean War, which elicited a strong feeling of compassion and existential thoughts about life (ID 199).

The codes indicating positive appraisals or “mixed/complex” feelings were not assigned to the responses of the remaining 10 participants in the sadly-moved class (40% of the participants this class). These cases might represent examples of the purely negative cases mentioned by Deonna [56]. Examination of the respective responses reveals that two cases were assigned codes such as “pleasant” feeling or “smiling.” Even though these codes were not included in the LCA and our classification of mixed cases, these two reports might therefore also be viewed as examples of mixed emotional experiences. The responses of the remaining eight participants did not include hints of positive appraisals or positive feelings. One example is a participant (ID 252) who received a phone call on Mother’s Day informing her that her mother had died in the psychiatric clinic where she had spent the last eleven years of her life. Another participant (ID 232) recalled how his sister, who was suffering from dementia, did not recognize him on the phone when he called to wish her a belated happy birthday.

### Latent classes of the “being-moved” prototype

As detailed when discussing the LCA model comparisons presented in Table 1, we also selected the two-class model as the best-fitting model for the “being-moved” prototype. In the following subsections, we present and compare these two classes. As the probability of code assignment was slightly to considerably higher for almost all codes (11 out of 14) in class 1 than in class 2, we labeled class 1 “extended description” and class 2 “basic description.” Results for an alternative three-class model for the prototype are reported in S3 Appendix.

#### The extended-description and basic-description classes

**Fig 3:**
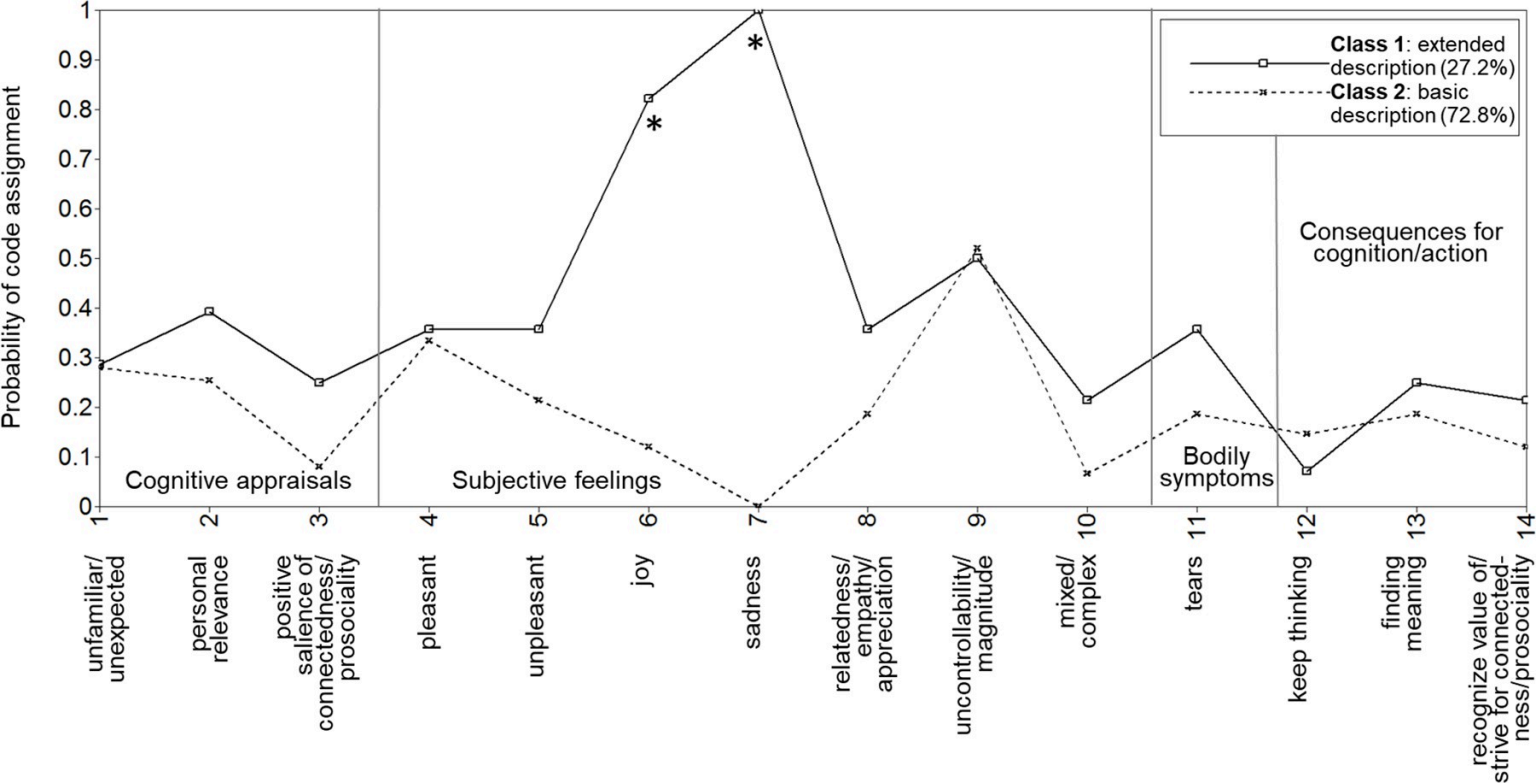
Latent classes for characteristics of “being moved” in descriptions of the “being-moved” prototype. * Significant difference between class 1 and class 2 at the Bonferroni-corrected alpha level of *p* < .0036.

The extracted two classes (Fig 3) differed in the average number of different codes applied (more codes in the extended-description class, *M*_class1_ = 9.5, than in the basic-description class 2, *M*_class2_ = 5.6; see Table 2). At the Bonferroni-corrected alpha level of *p* < .0036, only “joy,” *r*_*Grb*_ = .70, 95% CI [.51, .84], and “sadness,*” r*_*Grb*_ = 1.00 (note that the CI cannot be computed because of the perfect correlation, i.e., sadness codes were assigned only in class 1), codes were more likely to be applied in the extended-description class than in the basic-description class. Small differences for “positive salience of connectedness/prosociality,” *r*_*Grb*_ = .17, 95% CI [.02, .37], and “mixed/complex,” *r*_*Grb*_ = .15, 95% CI [.00, .34], failed to reach significance at the corrected alpha level.

Notably, the class membership for the personal experience and for the prototype were unrelated, χ^2^ (1) = 1.30, *p* = .255. Participants viewed “being moved” by positive or negative events as prototypical instances of this emotion, regardless of whether their own reported experience had been classified as joyfully moving or as sadly moving. Participants differed only with regard to whether they reported joy and sadness as prototypically associated feelings.

Importantly, these reports largely mirror the finding of separate joyfully-moved and sadly-moved classes for personal experiences. A few participants characterized “being moved” as a co-occurrence or blend of emotions, for example, “The feeling is close to sadness, yet it still has a hopeful, joyful component” (ID 206). However, the majority (20 participants, i.e., 87.0%) of the 23 participants whose responses included both joy and sadness as prototypical feelings said that one feels predominantly either joy or sadness: What “being moved” feels like is “very dependent on whether one is positively or negatively moved. If one is positively moved, one will feel joy, happiness, or also agitation; if negatively moved, then it will be sadness or also agitation” (ID 170).

#### Co-occurrence of positive and negative elements in descriptions of the “being-moved” prototype

As with the personal experiences, we were interested in a closer analysis of the co-occurrence of positive and negative appraisals and subjective feelings in the descriptions of the “being-moved” prototype. As the prototype of most participants featured discrete positive and negative types of “being moved” rather than a blend of positive and negative elements, we cannot consider responses featuring both positive and negative elements as descriptions of a “mixed” emotion. The “mixed/complex” code, which captures explicit characterizations of “being moved” as mixed or complex, was assigned to only roughly 11% of all descriptions of the “being-moved” prototype (see S1 Table).

In the extended-description class, 100% of the 28 participants reported both positive (“positive salience of connectedness/prosociality,” “pleasant,” or “joy”) and negative (“unpleasant” or “sadness”) elements, or alternatively, reported only positive or negative elements, but in addition characterized “being moved” explicitly as “mixed/complex.” In the basic-description class, 25.3% of the 75 reports featured such positive-negative/positive-mixed/negative-mixed co-occurrences. For 24.0% of the reports in the basic-description class, we assigned only positive codes, and one participant’s report (1.3%) was assigned only the “unpleasant” feeling code. The remaining half of the participants in the basic-description class (49.3%) did not define “being moved” by the selected positive and/or negative appraisals and feelings, which might suggest that they considered neither positive nor negative appraisals/feelings as a necessary feature of “being moved.” Rather, they characterized episodes of “being moved” as fully absorbing them or as intense, overwhelming, and uncontrollable emotional responses. Furthermore, felt importance, meaning, and insight were mentioned as prototypical features of “being moved” (roughly 19% of the participants in the basic-description class provided answers that were coded as “finding meaning”; see Table 2). For instance, one participant (ID 204) stated that one is moved when “in one moment you understand something, really understand something. . . become aware of something of which you were not aware before.” Other responses focused on feelings of “relatedness/empathy/appreciation” (again, roughly 19%; see Table 2), as exemplified by the following one-sentence definition: You feel moved when “you feel connected to the situation, the thought, the person” (ID 152).

## Discussion

We employed lay conceptions of the emotional state of “being moved” (“bewegt sein”) to answer our central research question: Does this term refer to a single emotion component profile or rather to different profiles with some overlap in terms of cognitive appraisals, subjective feelings, bodily symptoms, and consequences for thought and action? LCA provided evidence for two classes of profiles—being joyfully moved and being sadly moved—underlying participants’ descriptions of a moving personal experience. In the following, we not only summarize and discuss our empirical findings, but also propose a scientific construct of being moved that integrates both classes that are evident in the lay concept. We will argue that broadening our theoretical understanding is called for rather than accepting the notion that the lay concept of “being moved” is broader and more encompassing than our scientific construct of it. As stated by Reisenzein, “[T]heories of emotion are also supported by uncontroversial everyday facts about emotions they can explain, and are put into doubt if they fail to account for them” [28 p. 180]. Our findings suggest that, at least for German-speaking individuals, it is one such uncontroversial fact that the term “bewegt sein” designates both a joyful/positive and a sad/negative type.

In the joyfully-moved class, positive salience of connectedness/prosociality appraisals, feelings of joy and relatedness/empathy/appreciation, and a bodily sensation of warmth were reported more frequently than in the sadly-moved class. There was also a trend of more frequent positive salience of agency appraisals, which did, however, not reach significance after Bonferroni correction. Negative salience of agency as well as connectedness/prosociality appraisals and feelings of sadness were found more frequently in the sadly-moved class than in the joyfully-moved class (with a tendency to also report moist eyes/tears more frequently). In contrast, consequences for thought and action were not distinctive of class membership, except for a trend to keep thinking about joyfully-moving experiences more than about sadly-moving experiences. In both classes, a predominant consequence of “being moved” for thought and action was valuing and/or striving for connectedness/prosociality.

Descriptions of the “being-moved” prototype matched the finding of separate classes of joyfully-moving and sadly-moving experiences. Participants did not describe either being joyfully moved or being sadly moved as prototypical, but rather considered it typical for “being moved” that this emotion can have positive and/or negative elicitors. Participants in the two latent classes were distinguished only by whether they reported joy and sadness as typical subjective feelings associated with “being moved” (extended-description class) or not (basic-description class). While joy, sadness, and love already have been identified as central associated emotions in the conceptual structure of “being moved” [8, 9], our findings add to the literature by showing that the lay concept of “being moved” does not include the co-occurrence of joy and sadness as prototypical.

While these findings cannot settle ongoing debates on being moved, their integration with prior theorizing and research can inspire new answers to the two major questions brought up in the Introduction: (1) What elicitors and appraisals are characteristic of being moved? (2) What is the valence of being moved?

### What elicitors and appraisals are characteristic of being moved?

The core relational themes of being moved proposed in the literature focus on either the social elicitors of being moved [8, 9, 13–15] or on the salience of core values of any type [4, 5, 56]. Might the scientific construct of being moved therefore best be limited to the common ground shared by all proposals—being moved is elicited by social values—or does it need to be conceptualized more broadly?

Our present findings and previous studies [21, 23, 45] have revealed that relationship-related events, interactions with close others, and the affirmation of social values are particularly potent elicitors of “being moved.” Comparing the appraisals for agency and connectedness/prosociality, the latter stand out as being most frequently reported. Moreover, our LCAs did not produce a class with predominantly agency-related appraisals (even when considering up to four latent classes; see S2 Appendix). These findings provide additional support for the proposal that being moved is primarily defined by social connectedness and attachment [6, 9, 14, 15, 18, 58, 59].

However, it seems that defining being moved as an attachment emotion is not sufficient to distinguish it from other social emotions. This is not much of a concern within the kama muta framework, as the emotion designated by this artificial term might just as well be labeled as “being moved” as it might be labeled as “tenderness,” “sympathy,” “compassion,” “nostalgia,” and so forth [14, 15, 59]. Still, extant approaches to being moved and kama muta have proposed additional features that might distinguish being moved from related emotions. These include appraisals of suddenness, rapid temporal change, unexpectedness, figure-ground contrast (“standing out”), and surpassing a standard [4, 5, 15, 21, 45]. Other approaches have highlighted the apprehension of value and appraisals of meaning and truth [6–8, 52, 91, 92]. Our participants likewise reported the cognitive consequences of finding meaning and continuing to think about the moving experience as characteristic of both types of “being moved.” A distinguishing feature of being moved thus might be felt value and personal meaning.

Drawing on theories that are based on a hierarchical structure of goals and actions [93–96], we surmise that the felt meaning while being moved results from an abstract, high-level cognitive representation of ongoing events and actions as linked to the end goals of an individual’s striving. Experiences can be construed in different ways, ranging from concrete, contextual details of goals, events, and actions to abstract, decontextualized mental representations that convey the meanings of goals, events, and actions. Different motivational constructs, such as end/terminal goals, values, needs, and motives, have been employed to conceptualize the highest level of the goal hierarchy. As it is beyond the scope of this paper to define and distinguish these constructs, we will draw on Schwartz’s theory of basic human values [97, 98] for expository purposes. In this theory, values are defined as trans-situational goals, which are grounded in one or more universal requirements of human existence (i.e., needs) and serve as life-guiding principles.

The proposed hypothetical nexus of being moved and end goals does not imply that every end goal or end state within a goal and action hierarchy can elicit being moved. Rather, we posit, in accordance with previous theorizing, that communal sharing relationships [15, 99] and basic attachments [parent-infant, filial, and pair bonds; 60] constitute the developmental basis of being moved: Individuals can feel moved by communal sharing/attachment relationships, the values and ideals that have been adopted through socialization in these relationships, and other special objects, places, and abstract entities (such as “the nation”) to which they have formed extended attachments during their socialization [see also 6].

While many end goals are social in nature, there are exceptions. In Schwartz’s theory, values are grounded in three types of needs: “needs of individuals as biological organisms, requisites of coordinated social interaction, and survival and welfare needs of groups” [97 p. 4]. Values that stem from organismic needs, such as stimulation and hedonism, hardly have the potential to move people. This also holds true for end goals that serve a social distancing function, such as striving for autonomy and competition at the expense of social connectedness. However, this does not mean that people cannot be moved by achievement. The defining goal of the achievement value is striving for personal success through demonstrating competence in terms of prevailing social standards [97]. An achievement thus must be valuable to the society. It is precisely those moments when individual achievements are framed as achievements of relevance for a larger social group, society, or nation (e.g., through award ceremonies) that are often cited as moving.

In sum, we propose that being moved is elicited when social and socialized end goals are cognitively activated. Construal level theory [96] offers one explanation of what motivates more abstract, decontextualized mental representations when feeling moved: When events are perceived with greater psychological distance, this leads to more abstract and value-related cognition [100]. Psychological distance is defined as the subjective experience that something is far away from the self in the here and now. An object, action, or event can transcend the here and now in different distance dimensions, such as time (past and future events), space (distant objects, remote locations), social distance (another person’s perspective), and hypotheticality (what might have been, fictional events).

Several of our participants’ descriptions suggest that distancing mechanisms might have been operative in their moving experiences. “Being moved” was often felt by witnesses of rather than participants in an event [see also 9, 19, 22]. In our study, 48% of the participants reporting a sadly moving experience and 25% of those reporting a joyfully moving experience were not directly involved in the event. Taking a third-person (witness) as opposed to first-person (agent) perspective causes a more abstract construal of the same actions [101].

This explanation also fits well with reports of aesthetic experiences of art and nature as elicitors of “being moved.” The distancing-embracing model of the enjoyment of negative emotions in art reception [50] presents an account of how distancing mechanisms pave the way for being moved by artworks, most notably by artworks that also involve us in negative emotions.

The high-level construal characteristic of being moved may by itself contribute to this emotion’s positive appeal. Hong and Lee [102] demonstrated that individuals with high-level construals reacted as favorably or even more favorably to ads with mixed emotional content than to purely happy ads, in contrast to individuals with low-level construals who preferred happy ads over mixed-emotion ads. Williams et al. [103] showed that abstract as opposed to concrete thinking enhanced positive evaluations of both positive and negative experiences. A high-level, abstract construal of events while being moved might thus help to integrate negative affect into an overall positive experience.

### What is the valence of being moved?

To determine the valence of being moved, it will be helpful to consider that an emotion can have more than one valence, as is also suggested by the co-activation account of mixed emotions [cf. 61–63]. Specifically, we will build on the distinction between micro-valences and macro-valence [65]. Micro-valences are individual appraisals and the associated feelings that are specific to one component in the multi-component emotion network. For instance, at a funeral, an individual may mourn the loss of a loved one (a negative micro-valence) but also experience moments of mnemonic love for the deceased person (a positive micro-valence). Macro-valence is the integrated assessment of valence across all micro-valences. It serves as a common currency that allows one to evaluate and choose between situations and behaviors, for instance, to decide whether a funeral qua funeral was, overall, a positive experience, even though the death of a close person certainly does not have a positive valence.

All accounts of being moved [e.g., 4, 5, 9, 10, 15, 47, 49, 50], as well as our participants’ reports, agree that being moved is characterized by positive macro-valence. Macro-valence is directly linked to the resulting action tendency to approach or to avoid an emotion-eliciting experience. While our coding manual allowed for coding tendencies to avoid feeling “moved,” this code (822) was assigned to the moving personal experiences of only two participants (1.9%; see S1 Table). Thus, it might be misleading to call being moved a “mixed” emotion. Having mixed feelings implies ambivalence, which is associated with negative macro-valence [e.g., 104]. Being moved, in contrast, does not involve an approach-avoidance conflict [66], suggesting that it might be more appropriately labeled as a “blended” emotion that integrates positive and negative micro-valences into an overall desirable emotion.

To determine the extent to which “being moved” is a mixed/blended emotion, Cova and Deonna [4, 5] considered the valence of the eliciting event (evaluation of the event as positive and/or negative). They found that 19% of experiences of being “particularly moved” reported in their study were mixed, whereas 61% were “purely positive” and 17% “purely negative.” This led the authors to conclude that “situations that move us do not typically involve both negative and positive elements” [5 p. 360].

Our participants’ detailed descriptions of the appraisals and subjective feelings characteristic of “being moved” support a different estimate of the percentage of blended experiences. Positive or negative antidotes to the predominant appraisals and feelings or “mixed” feelings were reported by about 60% of the respondents. In line with the literature [4, 9–11, 19, 21–23], experiences in the joyfully-moved class often (64.2%) involved a positive foreground standing out against a negative background of appraised negative salience of connectedness/prosociality, negative salience of agency, and/or “mixed” feelings and feelings of sadness. Experiences in the sadly-moved class often (60.0%) had a negative foreground standing out against a positive background of appraised positive salience of connectedness/prosociality and/or agency or were characterized as “mixed/complex.”

Still, a substantial percentage of the participants’ responses included exclusively positive appraisals and emotions in the joyfully-moved class (32.1%) and exclusively negative appraisals and emotions in the sadly-moved class (40.0%). Whereas the former finding accords with the literature, the latter seems to contradict the notion that being moved is an overall pleasurable emotion that is distinct from pure sadness [4, 7–9, 14, 50, 66].

This raises two critical questions: (1) Should emotion components with a negative micro-valence be considered as part of the emotion being moved or rather as part of another emotion (sadness) [see 5]? Moreover, (2) if the answer to this question is that negative emotion components should be included in the construct of being moved, would this lead to the consequence that the macro-valence of being moved is negative in these cases?

Kama muta theory excludes negatively valenced components from the kama muta emotion [15, 18, 47]. Based on our conceptualization of emotion offered in the Introduction, it might be argued that the kama muta construct captures the non-verbal subjective feeling characteristic of being joyfully moved. Cova and Deonna similarly limited their construct of being moved to cases of being positively moved and mixed cases [4, 5]. They viewed cases of being negatively moved as instances of sadness and as overall unpleasant emotions (i.e., having a negative macro-valence).

In contrast to both the kama muta theory and Cova and Deonna’s account, we argue for the inclusion of being sadly moved and the associated negative emotion components in the construct of being moved. Limiting being moved to being joyfully moved is problematic for both pragmatic and theoretical reasons.

From a pragmatic perspective, such a limitation would cause a mismatch between the conceptual and operational definitions of being moved in future studies relying on self-reports of emotion based on the vernacular term “being moved.” Similar to other vernacular emotion terms that can be used to designate different types of an emotion, such as awe/sublimity [35, 105] and gratitude [36], we need to be aware that self-reports of “being moved” rely on the common-sense understanding of this term as including both a joyful and a sad type. If our empirical evidence for a type of “being moved” that is associated with sadness were rejected, future studies would need to operationalize being moved as the full emotion component profile of what we here report as the primarily joyful type of being moved. Researchers studying kama muta have already taken this approach and employed multi-item measures of several emotion components [17, 18]. However, it might not be feasible to employ such detailed measures in studies that seek to investigate and compare multiple emotions.

More importantly, there are also theoretical reasons for including negatively valenced components in the construct of being moved. The exclusion of emotion episodes with predominantly or even purely negative eliciting events from this construct seems to be based on the premise that negatively valenced events and appraisals cannot elicit emotions with positive macro-valence. In contrast to such an assumption, we propose that being moved is defined by its positive macro-valence, irrespective of whether the eliciting events are positive or negative. In fact, positive macro-valence may be an important determinant of whether an emotion is labeled as “being moved” rather than “sadness.” In line with this assumption, self-reports of “being moved” have been found to mediate the initially positive association between self-reported “sadness” and enjoying or wanting to see, read, or hear sad film clips [11, 19], poems [106], and music [107]. Once the overlap in the emotion components of “being moved” and “sadness” is statistically controlled for, only “being moved” is positively related to enjoyment, underscoring the positive macro-valence and the approach tendency that are characteristic of this emotion.

Can we assume that the “purely negative cases” of “being moved,” as described by Deonna [7, 56], also have positive macro-valence? According to Deonna, such cases “are simply cases of sadness or sorrow. They are the opposite of being moved in that they constitute the registering that a positive value (what is dear to us) has been vanquished by a negative value. Sadness is the experience of the loss or ruin of positive values” [56 p. 65]. Indeed, sadness is prototypically characterized as an emotion with negative macro-valence. It responds to irrevocable losses and to the non-attainment of goals and in the end helps individuals to disengage and let go [e.g., 33, 55, 108, 109].

Based on our data and theorizing, we disagree with the assumption that sadly moving experiences, even those where no positive elements in the eliciting situation are reported, are cases of pure sadness. If the hope to see a positive value emerge in a specific situation is thwarted and has to be given up, this leads to sadness. However, labeling the resulting emotion as “being moved” rather than simply “sadness” communicates an important difference between the two emotions: While the label “sadness” indicates a negative experience and a tendency to disengage from further pursuit of a valued goal or relationship, “being moved” indicates a meaningful experience that motivates continued engagement with and even fighting for a valued goal or relationship.

This argument also ties back to our previous proposal of characterizing being moved as elicited by abstract representations of social values and (extended) attachments. As such values and attachments are not “out there” in the world, but rather abstract concepts in our minds, they cannot be lost or ruined during events that pose threats to their actualization. Even in such situations, we can cognitively access our values and representations of security- and meaning-providing close others. While specific events might be incompatible with our values, and close others might leave and even die, our abstract representations of the respective values and attachments not only outlive these events, but can even be particularly strongly activated in such negative circumstances.

In accordance with this view, the literature on “threat-compensation” [110]/“violation-compensation” [111] has highlighted that threats to a sense of meaning, attachment security and social integration, certainty and personal control, self-esteem, faith in the social system, symbolic immortality, and so on all elicit a negative arousal state that prompts a reactive/defensive approach motivation [110–115]. This motivation can lead to various action tendencies, such as an increased desire for social affiliation, heightened identification with a social group, or affirmation of abstract ideals and values [113]. Abstract values are a highly efficient focus for reactive approach motivation [114], especially in situations that do not offer options for other behaviors. They can be approached by heightening imagined commitment to them and in this capacity are resistant to temporal frustration or disillusionment. As affect serves to embody abstract human values [116, 117], it is possible that the mere cognitive activation of a value triggers the associated feelings of reward.

The literature on threat-compensation has linked the experience of threat to a higher level of action identification [118] as well as to the activation of mental representations of attachment figures [119]. The attachment literature has similarly highlighted the calming and soothing effect of the symbolic availability of internalized attachment figures. Simply thinking about such figures results in an “infusion of positive affect” [84 p. 61].

Thus, even seemingly purely negative cases of “being moved” that make social values or relationships salient in a negative way may well produce particular kinds of self-rewarding, meaningful feelings that are associated with a given value or relationship. As characterized by Deonna [56 p. 66], being moved “is an unsettling yet soothing form of pleasureless contentment brought about by the contemplation of a specific core positive value making a stand.” Mori and Iwanaga [49] have likewise conceptualized being moved as resulting from the emergence of life-guiding ideas, which leads to phasic physiological calming in a state of tonic physiological arousal. Importantly, this may hold true even when the positive value or life-guiding idea is actually compromised in a given situation and manages to “make a stand” only in the individual’s mind.

The conclusion that felt meaning is independent of the valence of eliciting events is further supported by findings reported by Murphy and Bastian [120]: Both extremely painful and extremely pleasant life events were perceived as more meaningful than less extreme events. This effect was partly explained by the greater emotional intensity of extreme events and their capacity to induce contemplation.

It therefore seems likely that languages (or at least the German language) would include emotion terms that designate such intense feelings of meaningfulness irrespective of the valence of the eliciting event and that “being moved” is a highly prototypical case of such an emotion term. Prior to concluding with our resulting theoretical construct of being moved, we would first like to acknowledge some limitations of our study.

### Limitations

The most important limitation of our findings might be that they first and foremost apply only to the German vernacular label for “being moved” (“bewegt sein”). We do not know whether these findings generalize to the many other languages that have verbal labels for “being moved,” such as English, French, Spanish, Italian, Dutch, Norwegian, Finnish, Hungarian, Polish, Serbian, Russian, Turkish, Japanese, Mandarin, and Hebrew [cf. 8, 13, 17, 18]. It would be interesting to determine whether the vernacular labels in other languages also include a joyful and a sad type. Extant publications suggest that this is the case, at least for English and French [4, 5], Dutch [21], and Japanese [10].

Another limitation is that the reports of “being moved” were retrospective. Our participants’ recollections of the experience might have been selective and driven by their concept of “being moved.” For instance, whereas crying and “being moved” are strongly associated for laypersons (consider the formulation “moved to tears”), goosebumps might be less readily associated with “being moved” and less well remembered than tears. It is possible that less prototypical and memorable emotion components, such as goosebumps/chills, were underreported in our study.

As we asked our participants to list all emotion components of “being moved,” we cannot determine which components were more or less central to categorizing the experience as “being moved” or which components were weighted more or less heavily when determining the emotion’s macro-valence. Some appraisals, such as “moral goodness” [see 65 p. 9], might be more important for both labeling emotions and determining macro-valence than others. Considering that appraisals of compatibility with social norms and self-ideals [9] and of morality [16, 58] have received high ratings in prior studies on “being moved,” it seems likely that felt moral significance is among the most characteristic appraisals of being moved. It would be interesting to investigate in future research which emotion components are given the greatest weight when people choose whether to label an experience as “being moved” or rather use a different label.

We would also like to acknowledge that much of the cited research was published after we completed the development of our coding manual in 2014. While more recent publications have led to many important insights on being moved, our central questions of whether being moved is limited to social elicitors and whether there are different types of “being moved” were already raised in the first publications on the topic [4, 8–10] and informed the present research. We believe that it speaks to the validity of our coding that we had already included codes for connectedness and prosociality before kama muta research highlighted the importance of closeness appraisals [13, 15, 16, 47, 58].

Our sample size of 106 is smaller than some of the recommendations found in the LCA literature, such as a desirable *N* of at least 300 [43]. Having too small a sample might lead to choosing too few classes [cf. 43, 78]. However, as stated in the Methods section, it is erroneous to assume that we can determine a minimum required sample size that would fit for all studies. Simulation studies have shown that when there are two well-separated latent classes, sample sizes below 50 may be sufficient [43, 78, 79]. Therefore, we conclude that we have presented solid evidence for at least two latent classes of both moving experiences and prototypical descriptions of “being moved.” We cannot rule out the possibility that future research with larger samples might find evidence for more than two classes. Moreover, as the class sizes were uneven, the power to detect significant differences between code assignment in the two classes was limited to medium-sized to large effects. It is therefore possible that additional differences between the two types of “being moved” will be found in future studies. Likely findings might be that respondents are less often directly involved in sadly moving events and that codes indicating “positive salience of agency” are less frequent for negatively moving experiences. We note that more frequent reports of tears (and less frequent reports of warmth) have already been found to distinguish between being negatively moved and being positively moved [5].

Finally, we employed a convenience sample that consisted largely of highly educated women. It should be noted, however, that we recruited participants for the studies of our research cluster by various means, including advertisements in the Berlin underground. Our sample is therefore more diverse (e.g., in terms of age) than the psychology student samples on which many studies have relied.

### Conclusion

Having integrated the findings of our study with prior theorizing and evidence, we would like to draw some conclusions for future research. Personal experiences of “being moved” and descriptions of the “being-moved” prototype revealed two types of “being moved” with different emotion component profiles. The first was elicited by positive events that rendered social attachments and values highly salient through their (often unexpected and unlikely) actualization. The fulfillment of social needs, high compatibility with moral standards, and associated pleasant feelings are part of the component networks of both joy and “being moved,” which is why this type was labeled “being joyfully moved.” A second, generally less frequent type of “being moved” was elicited by negative events, during which social attachments and values became highly salient because they were not actualized in the situation. Severed attachments, frustrated social needs, and violated values account for an overlap in the emotion networks of sadness and “being moved,” thus representing a case of “being sadly moved.”

Nevertheless, both types of “being moved” can be considered as positive emotions at the macro-valence level, even if especially the sadly-moved type is often blended with negative elements at the micro-valence level. In some episodes of “being moved” that are at first glance purely negative, the positive element might be limited to the cognitive activation of meaning-providing values, ideals, and attachments. The low frequency of such apparently purely negative cases of “being moved” in the relevant studies, including the present one, might reflect a greater difficulty in retrieving from memory positive implications coming from situations that do not provide any obvious cues for positive values and attachments. Moreover, both types of “being moved” were reported to have the same consequences for thought and action: cognitive engagement with the event and finding meaning as well as an increased valuation of and striving for connectedness and prosociality.

Based on these findings, we developed a theoretical concept of being moved that is informed by core themes and functions that also seem to guide laypersons’ labeling of the emotion. We have proposed that the central elicitor of being moved is the cognitive activation of social and socialized end goals. Being moved means feeling the goodness of individuals, social entities and objects, and abstract values and ideals as sources of the final/ultimate meaning in a person’s life. This feeling is highly rewarding, which explains why people feel attracted to moving experiences, even when they include negative elicitors.

## Supporting information

S1 Appendix Questionnaire on “being moved”. Presents an English translation of all instructions and questions about “being moved” that were asked in the online study.
(DOCX)

S2 Appendix Moving personal experiences: Alternative models. For comparison purposes, this appendix presents findings for two alternative LCA models for moving personal experiences: a four-class model with 16 indicators and a two-class model with 20 indicators.
(DOCX)

S3 Appendix The “being-moved” prototype: Alternative models. For comparison purposes, this appendix presents findings for two alternative LCA models for the “being-moved” prototype: a three-class model with 14 indicators and a two-class model with 20 indicators.
(DOCX)

S1 Table Overview of codes, code examples, and code frequencies. Presents English translations of all code categories included in the German coding manual together with exemplary answers and frequencies for each code.
(DOCX)
