# Supplementary material for: Lay conceptions of “being moved” (“bewegt sein”) include a joyful and a sad type: Implications for theory and research
Source: PLoS One. 2022 Oct 27;17(10):e0276808. doi: 10.1371/journal.pone.0276808 (PMC9612584; doi:10.1371/journal.pone.0276808)
Supplement: S1 Appendix — Presents an English translation of all instructions and questions about “being moved” that were asked in the online study. (DOCX) [file pone.0276808.s001.docx]

**S1 Appendix**

**Questionnaire on “being moved”**

*Being Moved: Personal Experience*

Please remember moments when you felt moved. Think back to the last few months. Were there any situations that you experienced as moving? If you can think of several moving moments, please choose a particularly typical one. It is important that you would most likely describe yourself as “moved” at the chosen moment.

Now try to remember this moving moment—and how you felt—as precisely as possible. You may take a few minutes to do so. Then please go to the next page, where we ask you for a description of this moment.

Please also answer the questions on the following pages with regard to this moment. We would appreciate it if you could answer the questions as precisely as possible. Please describe everything in such a way that someone without your background knowledge can understand it.

[next page]

Please describe the moving moment.

1. When did you experience this moment?

2. Where were you? What situation were you in?

3. What happened at that moment?

[next page]

4. What was personally moving for you at that moment? What triggered this feeling? What impressions and thoughts about the situation contributed to eliciting this feeling?

[next page]

5. Please describe in more detail how it felt for you to be personally moved. How can one imagine your emotion?

[next page]

6. How did it physically feel to be moved? What physical sensations, reactions, or expressions accompanied your feeling?

[next page]

7. What further thoughts did feeling moved elicit in you? In what way did this experience influence your subsequent actions and thoughts?

[next page]

8. How moved were you during the situation just described? (rating: 1 *very little* to 5 *very strongly*)

[next page]

*Being Moved: General Description*

Now we are interested in the general characteristics and indicators of the feeling of being moved. Beyond a very specific moment or your own experience, what do you think it generally means to be moved?

Imagine explaining what being moved feels like to someone who has never experienced it. When answering the questions on the following pages, please also write down what you consider obvious and self-evident. At the same time, focus on the essential features that characterize this feeling.

[next page]

1. What characterizes the feeling of being moved? What does a moved person feel and think? How does she or he act?

[next page]

You have now thought a lot about what it means to be moved. Finally, we are interested in how you would describe the eliciting situation in general terms using one sentence.

The following example is intended to illustrate what this is about. If you had to complete the sentence “One gets angry if…” you could write, for instance, “… one perceives a behavior or event as an offense to oneself or to that which one cares about.”

2. Please try to describe in a similar way what moves people.

One feels moved if…
