# Supplementary material for: Lay conceptions of “being moved” (“bewegt sein”) include a joyful and a sad type: Implications for theory and research
Source: PLoS One. 2022 Oct 27;17(10):e0276808. doi: 10.1371/journal.pone.0276808 (PMC9612584; doi:10.1371/journal.pone.0276808)
Supplement: S2 Appendix — For comparison purposes, this appendix presents findings for two alternative LCA models for moving personal experiences: a four-class model with 16 indicators and a two-class model with 20 indicators. (DOCX) [file pone.0276808.s002.docx]

**S2 Appendix**

**Moving personal experiences: Alternative models**

In order to determine to what extent our results might depend on our decision to run a two-class LCA with 16 indicators for moving personal experiences, we also ran (1) a four-class LCA with 16 indicators (S2 Fig 1) and (2) a two-class LCA with 20 indicators (S2 Fig 2) for purposes of comparison. As can be seen in in S2 Fig 1, the sadly-moved class (class 1) was also present and basically unchanged in the four-class solution. The joyfully-moved class was split up into three sub-classes that showed a somewhat similar profile, but with a comparably low, intermediate, and high probability of being assigned the codes that are characteristic of the joyfully-moved class (i.e., “positive salience of connectedness/ prosociality,” “joy,” “relatedness/empathy/appreciation,” and “warmth”). In addition to differences in average probabilities, some codes were more prevalent in one of the three sub-classes than in the others. Specifically, there was one class (class 2) with a high probability of “connectedness/prosociality” appraisals, feelings of “relatedness/empathy/appreciation,” and a tendency to “recognize the value of/strive for connectedness/prosociality.” As a sub-class of the joyfully-moved class, we labeled this class “joyful/social.” Participants in class 3 focused on the mixed affective nature of “being moved,” as expressed by a combination of “joy,” “sadness,” “relatedness/empathy/ appreciation,” “uncontrollability/magnitude,” “mixed/complex,” “tears,” and “warmth” codes. This class therefore was called “joyful/ mixed.” The final class 4 was labeled “joyful/low,” because it resembles the profile of the joyfully-moved class, but the probability of code assignment never clearly exceeded 0.5 (“joy” was the most likely code in this class).

When we increased the number of indicators to 20 and ran a two-class model, we still found a joyfully-moved (class 2) and a sadly-moved (class 1) class (S2 Fig 2). The fit statistics for this model were highly similar to the model with 16 indicators presented in the paper: entropy = .864, *AvePP*_class1_ = .915 and *AvePP*_class2_ = .977, Vuong-Lo-Mendell-Rubin LRT *p* = .070 and adjusted *p* = .073, parametric bootstrapped LRT *p* = .000. Note that AIC = 2596, BIC = 2705, and sample-size adjusted BIC = 2576, but these numbers cannot be compared with the model with 16 indicators, as they necessarily are higher with more indicators. The proportions of the two classes were almost unchanged. Only three participants’ class assignment had changed: they had been in the joyfully-moved class and were now in the sadly-moved class. The four additional indicators “paying attention” (code 52012), “smiling” (code 6131), “muscle tension” (code 6091), and “personal improvement” (code 771) did not add much to distinguishing between the classes.

**S2 Fig 1. Latent classes for characteristics of “being moved” when describing an own experience:**

**Alternative 16-indicator four-class solution.**
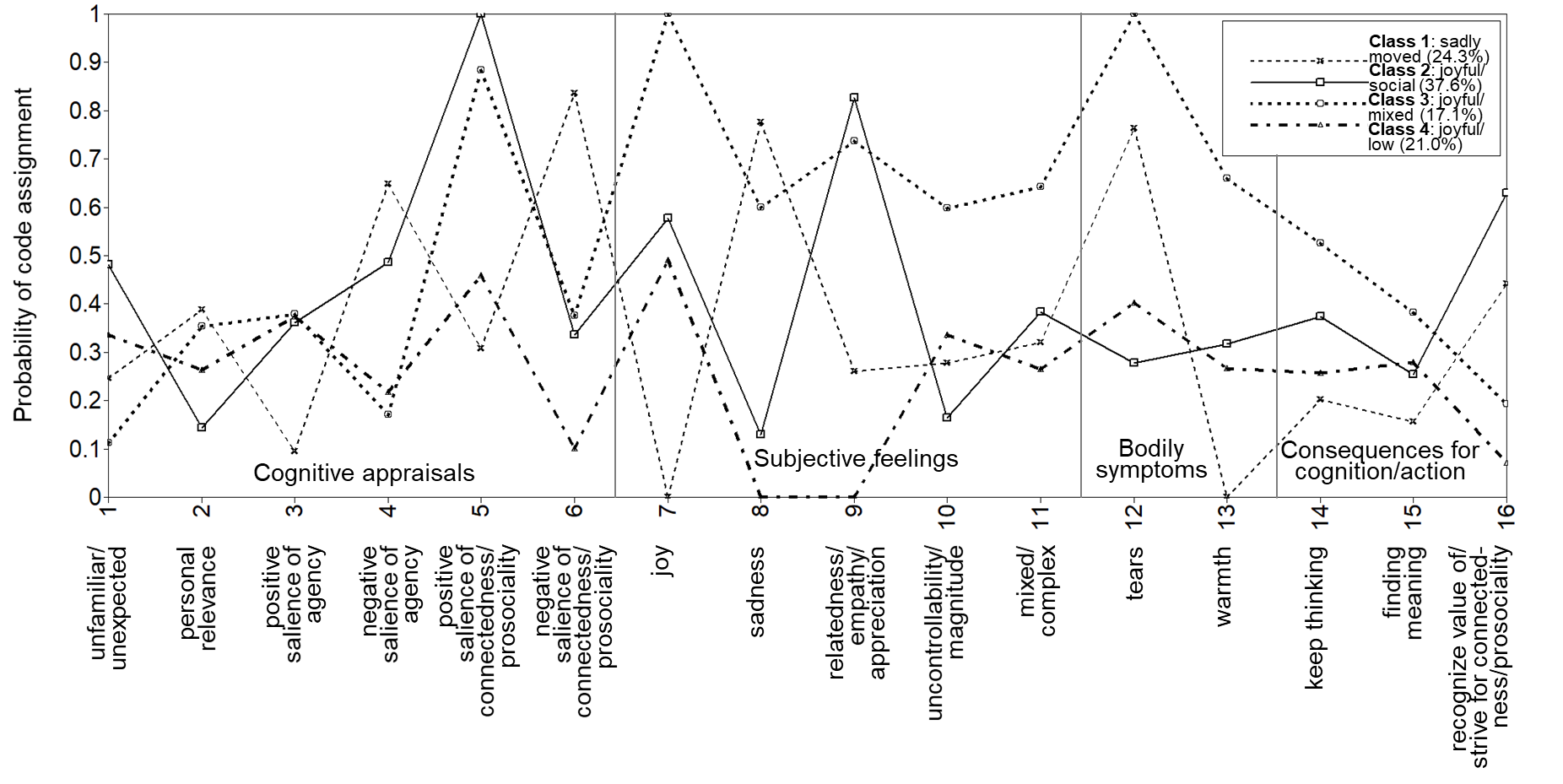


**S2 Fig 2. Latent classes for characteristics of “being moved” when describing an own experience:**

**Alternative 20-indicator two-class solution.
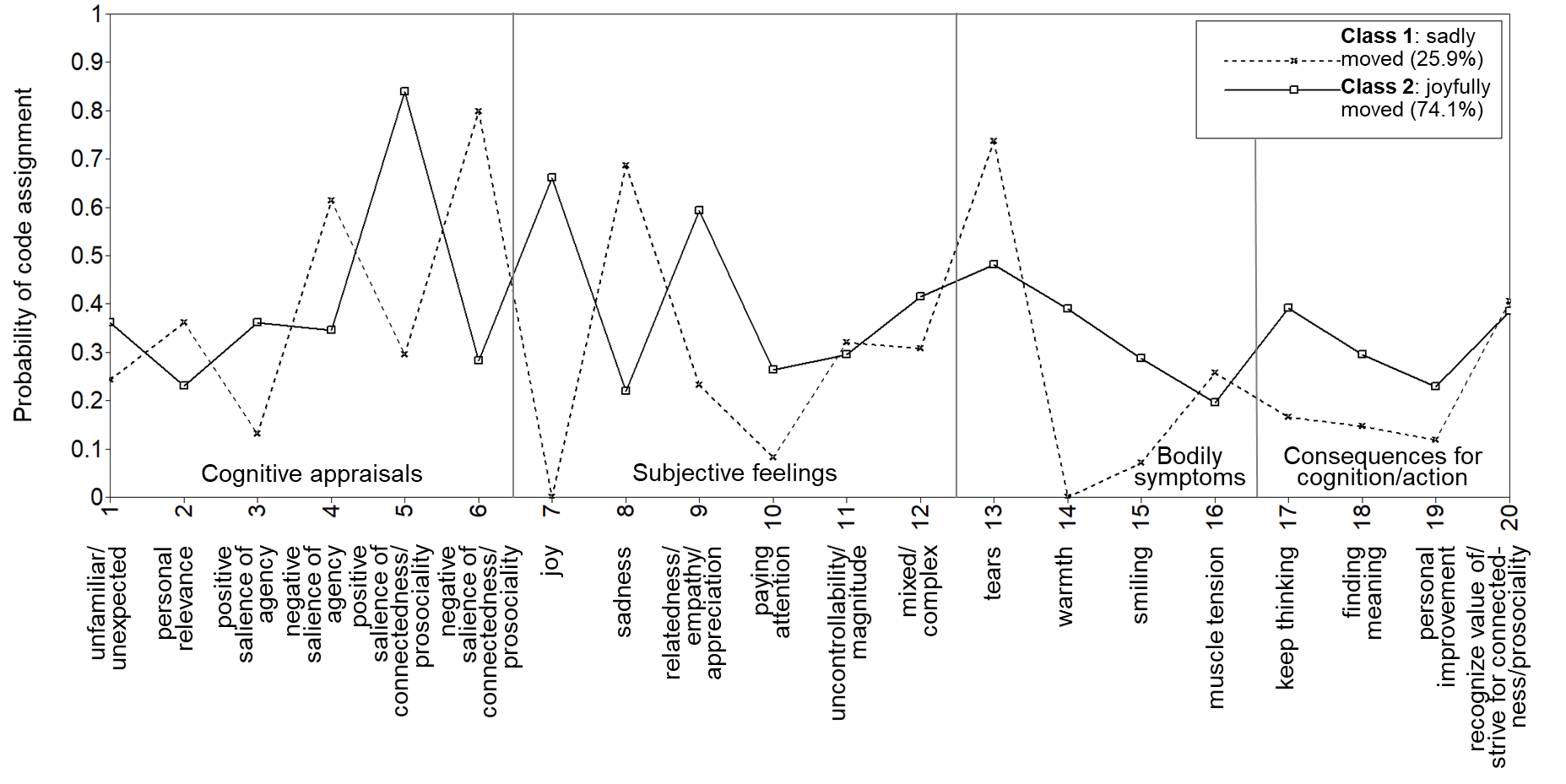
**
