# Supplementary material for: Lay conceptions of “being moved” (“bewegt sein”) include a joyful and a sad type: Implications for theory and research
Source: PLoS One. 2022 Oct 27;17(10):e0276808. doi: 10.1371/journal.pone.0276808 (PMC9612584; doi:10.1371/journal.pone.0276808)

**S3 Appendix**

**The “being-moved” prototype: Alternative models**

In order to determine to what extent our results might depend on our decision to run a two-class LCA with 14 indicators for the “being-moved” prototype, we also ran (1) a three-class LCA with 14 indicators (S3 Fig 1) and (2) a two-class LCA with 20 indicators (S3 Fig 2) for purposes of comparison. As can be seen in S3 Fig 1, the basic-description class gets split up into a class 3 with an overall low probability of code assignment and a class 2 with very high probabilities for the “pleasant” and “unpleasant” codes together with “uncontrollability/magnitude.” As a result, there is one class (class 1) focusing specifically on “joy” and “sadness” as prototypical feelings associated with “being moved” and a second class focusing on the positive and negative elicitors and/or feeling quality of “being moved” without reporting joy and sadness. Proceeding to four classes, we still did not obtain a class of participants who would define “being moved” as a purely pleasant, joyful emotion. Rather, participants in the fourth class emphasized the social nature of being moved, as expressed by high probabilities of the codes “positive salience of connectedness/prosociality,” “relatedness/empathy/appreciation,” and “recognize value of/strive for connectedness/prosociality” (together with “pleasant,” “unpleasant,” “joy,” and “sadness”).

When we increased the number of indicators to 20 and ran a two-class model, we still found a basic-description (class 2) and an extended-description (class 1) class (S3 Fig 2). The fit statistics for this model were slightly worse compared with the model with 14 indicators presented in the paper: entropy = .939, *AvePP*_class1_ = .968 and *AvePP*_class2_ = .997, Vuong-Lo-Mendell-Rubin LRT *p* = .130 and adjusted *p* = .135, parametric bootstrapped LRT *p* = .000. This likely is related to adding six indicators, “positive salience of agency” (code 4411), “negative salience of agency” (code 4412), “paying attention” (code 52012), “intense experience” (code 51051), “warmth” (code 6011), and “smiling” (code 6131), which did not contribute to distinguishing between the classes. Note that AIC = 1956, BIC = 2064, and sample-size adjusted BIC = 1935, but these numbers cannot be compared with the model with 14 indicators, as they necessarily are higher with more indicators. The proportions of the two classes basically were unchanged. Only one participant who had been in the basic-description class was now in the extended-description class.

**S3 Fig 1. Latent classes for characteristics of “being moved” in descriptions of the “being-moved” prototype:**

**Alternative 14-indicator three-class solution.**


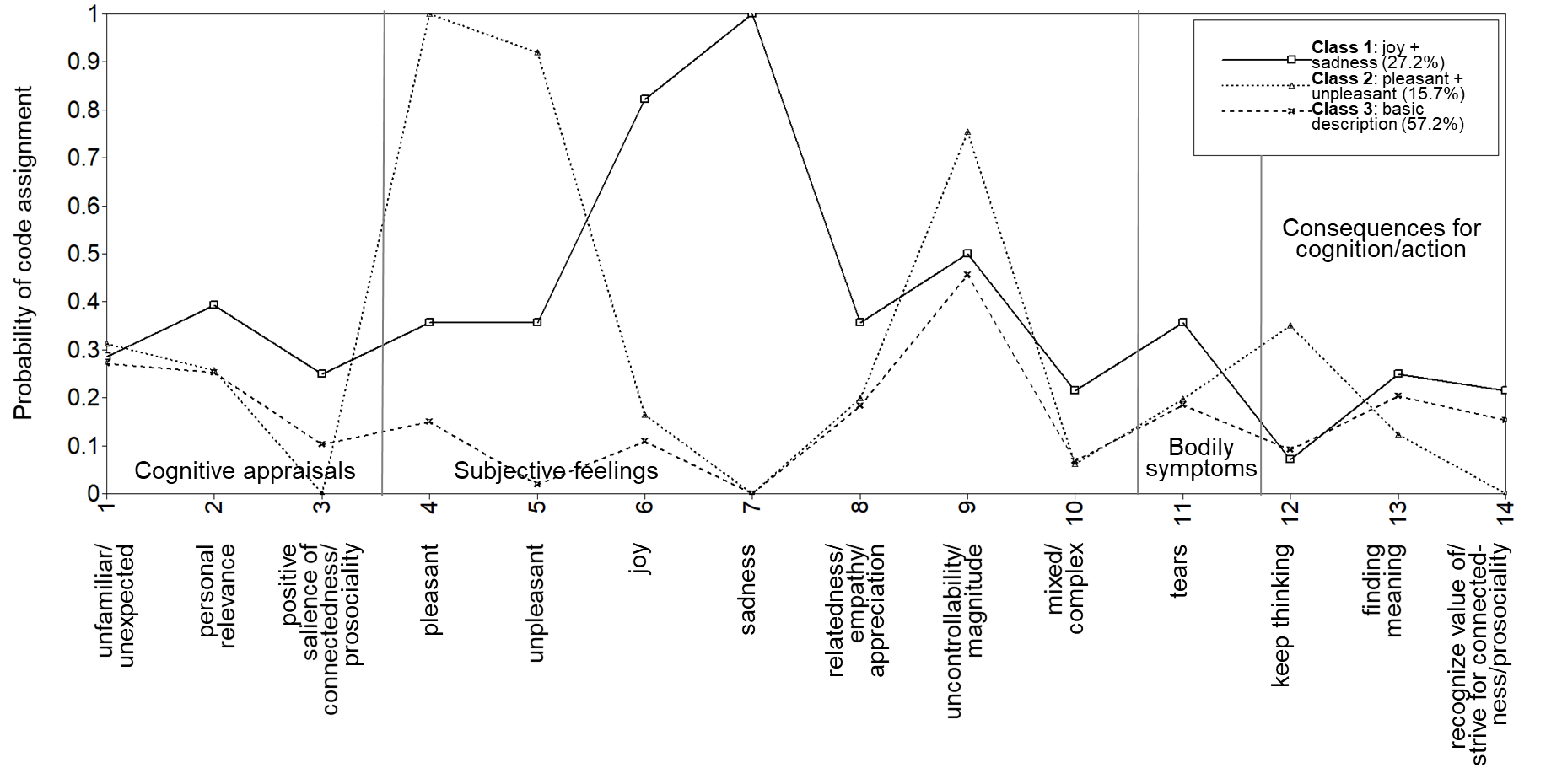


**S3 Fig 2. Latent classes for characteristics of “being moved” in descriptions of the “being-moved” prototype:**

**Alternative 20-indicator two-class solution.**


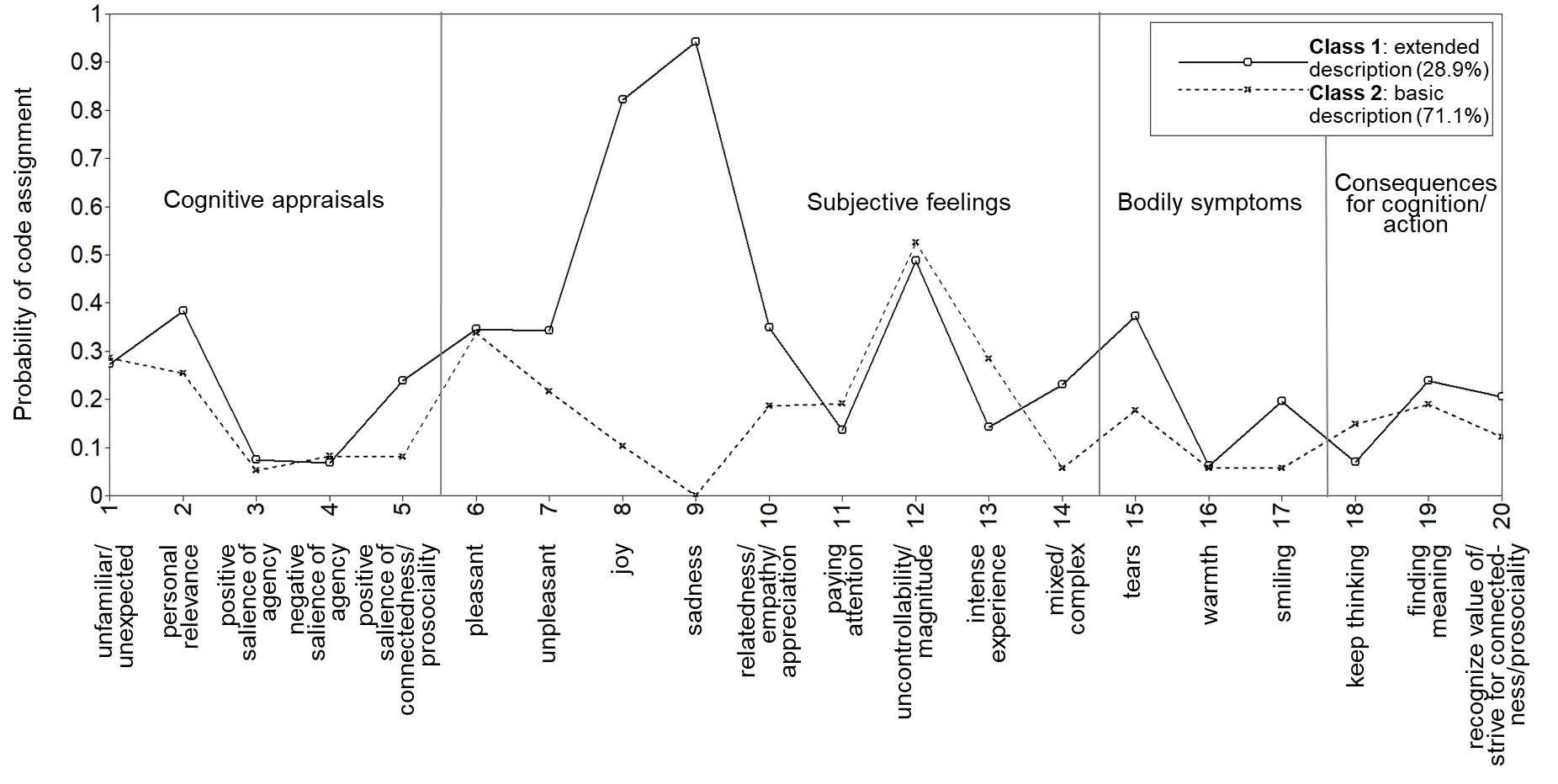

Supplement: S3 Appendix — For comparison purposes, this appendix presents findings for two alternative LCA models for the “being-moved” prototype: a three-class model with 14 indicators and a two-class model with 20 indicators. (DOCX) [file pone.0276808.s003.docx]
