# Supplementary material for: Lay conceptions of “being moved” (“bewegt sein”) include a joyful and a sad type: Implications for theory and research
Source: PLoS One. 2022 Oct 27;17(10):e0276808. doi: 10.1371/journal.pone.0276808 (PMC9612584; doi:10.1371/journal.pone.0276808)
Supplement: S1 Table — Presents English translations of all code categories included in the German coding manual together with exemplary answers and frequencies for each code. (DOCX) [file pone.0276808.s004.docx]

**S1 Table. Overview of Codes, Code Examples, and Code Frequencies (*N* = 106).**

|  |  |  |  |  | **Moving personal experience** | |  | **Being-moved prototype^a^** | |
| --- | --- | --- | --- | --- | --- | --- | --- | --- | --- |
| **Number** | **Label** | **Description** | **Examples from study^b^** |  | ***n*** | **%** |  | ***n*** | **%** |
| ***1 Time of event^c^*** | | | | | | | | | |
| 11 | ≤ 1 week | Event happened within the last week | today; yesterday; five days ago; last Saturday; a few days ago; about one week ago |  | 17 | 17.5 |  | - | - |
| 12 | ≤ 1 month | Event happened more than a week ago but within the last month | two weeks ago; about two to three weeks ago; a few weeks ago; four weeks ago; middle of February |  | 26 | 26.8 |  | - | - |
| 13 | ≤ 6 months | Event happened more than a month ago but within the last six months | two months ago; about two to three months ago; it was a few months ago; March 1, 2013; middle of December last year; in September 2012 |  | 33 | 34.0 |  | - | - |
| 14 | ≤ 1 year | Event happened more than six months ago but within the last year | about eight months ago; October 4, 2012; in 2012; one year ago; about one year ago |  | 7 | 7.2 |  | - | - |
| 15 | ≤ 2 years | Event happened more than a year ago but within the last two years | New Year’s Eve 2012; about 1.5 years ago |  | 2 | 2.1 |  | - | - |
| 16 | > 2 years | Event happened more than two years ago | 2010; a few years ago; July 1, 2008; about five years ago; April 22, 2005; ten years ago; after the fall of the Berlin Wall; on Mother’s Day 1968 |  | 11 | 11.3 |  | - | - |
| ***2 Eliciting event*** | | | | | | | | | |
| *21 Person-related elicitors* | | | | | | | | | |
| 2111 | beginning of life | elicitors related to the beginning of life such as pregnancy, birth, or newborns; life after death; existence per se | giving birth; witnessing the birth of my daughter; touching a pregnant friend’s belly and feeling the baby move; holding a shivering emu chick |  | 6 | 5.7 |  | 2 | 1.9 |
| 2112 | end of life | elicitors related to the end of life such as dying, death, funerals, or memorial services for the dead; annihilation; mortality per se | funeral of my grandmother; receiving news of my grandmother’s death; embracing my dying father for the last time; telling my terminally ill mother it’s ok to die; experiencing the death of a nursing home patient as a temporary staff member; talking to my grandmother who recalled the death of her pregnant friend; death of my guinea pig; receiving news that my favorite dog had to be put down; experiencing the crucifixion scene during a live performance of Bach’s St. John Passion |  | 14 | 13.2 |  | 4 | 3.9 |

**S1 Table** *continued.*

|  |  |  |  |  | **Moving personal experience** | |  | **Being-moved prototype^a^** | |
| --- | --- | --- | --- | --- | --- | --- | --- | --- | --- |
| **Number** | **Label** | **Description** | **Examples from study^b^** |  | ***n*** | **%** |  | ***n*** | **%** |
| 2121 | positive turning points | elicitors related to positively experienced turning points in one’s own life such as a new life phase, experiencing something for the first time, or new possibilities opening up; general appreciation that life keeps changing | first time celebrating Christmas with my parents not at their home, but at my home; finally feeling at home after moving to a new city; receiving news that a publisher has accepted my manuscript; passing my final oral exam |  | 4 | 3.8 |  | 0 | 0.0 |
| 2122 | negative turning points | elicitors related to negatively experienced turning points in one’s own life such as termination of a life phase or experiencing something for the last time; general regret that nothing in life lasts forever | receiving a letter containing bad news |  | 1 | 0.9 |  | 0 | 0.0 |
| 2131 | recovery/end of suffering | elicitors related to recovery from physical or mental illness, overcoming disability, or successful therapy | receiving news that my grandmother’s heart surgery was successful |  | 1 | 0.9 |  | 1 | 1.0 |
| 2132 | illness/ suffering | elicitors related to physical or mental illness, suffering, disability, or addiction | one of my best friends telling me he has inoperable cancer; my daughter almost dying after she had an emergency caesarean; my boyfriend telling me he had planned to commit suicide; my grandmother telling me she was unable to sleep because of my grandfather’s illness and pain |  | 4 | 3.8 |  | 1 | 1.0 |
| 2141 | mastery/ competence | elicitors related to mastering challenges, achieving against the odds or beyond expectations, overcoming difficulties, or achievements being recognized or praised | having finished my trade test; handing in my diploma thesis; having to find my way without a map during a one-week hike in South Africa; after playing in a soccer match that ended in a draw; practicing reading with my first grader |  | 7 | 6.6 |  | 1 | 1.0 |

**S1 Table** *continued.*

|  |  |  |  |  | **Moving personal experience** | |  | **Being-moved prototype^a^** | |
| --- | --- | --- | --- | --- | --- | --- | --- | --- | --- |
| **Number** | **Label** | **Description** | **Examples from study^b^** |  | ***n*** | **%** |  | ***n*** | **%** |
| 2142 | helplessness/ incompetence | elicitors related to not being able to handle challenges, being helpless, inability/incompetence, or performance being criticized or considered insufficient | - |  | 0 | 0.0 |  | 0 | 0.0 |
| 2151 | strokes of luck/ wonders | elicitors related to having luck or experiencing something as a wonder | - |  | 0 | 0.0 |  | 0 | 0.0 |
| 2152 | catas­tro­phes/ misfortune/ accidents | elicitors related to natural catastrophes, accidents, famines, or poverty | - |  | 0 | 0.0 |  | 0 | 0.0 |
| *22 Relationship-related elicitors* | | | | | | | | | |
| 2211 | beginning of/ positive turn in relationships | elicitors related to starting, intensifying, or reestablishing a relationship such as engagement, marriage, moving in together, reunion, getting back together, or reconciliation; being accepted into a group | seeing my little sister after spending four months abroad; reunion with my cousin after 20 years; being a guest at a close friend’s wedding; my sister telling me that she got together with one of her friends; getting back together with my ex-husband; improving my negative opinion of a pupil after hearing from a colleague that she had praised my teaching; watching an online video (John Lewis’ Christmas advert “The Bear and the Hare”) |  | 11 | 10.4 |  | 4 | 3.9 |
| 2212 | end of/ negative turn in relationships | elicitors related to ending a relationship, reduced closeness, or a deteriorating relationship such as a farewell, temporary or permanent separation, or divorce; being excluded from a group | saying goodbye to my husband at the airport; at the farewell brunch of a youth exchange program; my girlfriend breaking up with me; my apartment mate telling me she wants to move out; telling my father he should at least say he feels sorry for leaving my mother; my sister suffering from dementia not recognizing my voice on the phone; watching the film “Jenseits der Mauern” in the cinema |  | 8 | 7.5 |  | 0 | 0.0 |

**S1 Table** *continued.*

|  |  |  |  |  | **Moving personal experience** | |  | **Being-moved prototype^a^** | |
| --- | --- | --- | --- | --- | --- | --- | --- | --- | --- |
| **Number** | **Label** | **Description** | **Examples from study^b^** |  | ***n*** | **%** |  | ***n*** | **%** |
| 2221 | positive experience of relationships/ good relationship quality | elicitors related to the experience of positive relationship quality, closeness, or mutual liking and respect such as caring, doing something special for another person, or feeling understood; acting and being prosocial in general | my mother organized a surprise party to welcome me back home after a stay abroad; my boyfriend driving with me to an animal shelter so that I could pick out a cat as a surprise present for my birthday; dancing the Viennese waltz with my boyfriend during dancing class (to the “Hochzeitswalzer” [wedding waltz]); my brother reciting a poem that he had written for my birthday; watching how my daughter interacts with older people at her work at a retirement home; my grandmother crying from joy when I told her I had been accepted for training as a physiotherapist; my father telling me he is sorry for what he said about my sister last evening; a patient at a forensic clinic, where I am doing an internship, wishing me a nice evening |  | 26 | 24.5 |  | 0 | 0.0 |
| 2222 | negative experience of relationships/ poor relationship quality | elicitors related to the experience of poor relationship quality, disliking, and disrespect such as conflicts, having a fight, mistrust, or complicated relationships; being mistrusting and rejecting in general | losing faith in the trustworthiness of my girlfriend; being ignored by my stepmother when trying to resolve an argument that we had |  | 2 | 1.9 |  | 0 | 0.0 |
| *23 Community-related elicitors* | | | | | | | | | |
| 2311 | collective recognition | elicitors related to public or collective recognition such as awards or graduation ceremonies, rituals to celebrate group admission, acceptance speeches, or monuments to honor someone | - |  | 0 | 0.0 |  | 0 | 0.0 |

**S1 Table** *continued.*

|  |  |  |  |  | **Moving personal experience** | |  | **Being-moved prototype^a^** | |
| --- | --- | --- | --- | --- | --- | --- | --- | --- | --- |
| **Number** | **Label** | **Description** | **Examples from study^b^** |  | ***n*** | **%** |  | ***n*** | **%** |
| 2312 | collective disrespect | elicitors related to public or collective disrespect such as rituals to expel someone from a group, withdrawal of rights or status, making misconduct public, or mobbing in public | realizing that I should leave the company I am working for because of their unfair treatment of and disrespect toward employees |  | 1 | 0.9 |  | 0 | 0.0 |
| 2321 | positive turning points | elicitors related to positive turning points at a societal, political, or cultural level such as a change of government, overturning of an unjust regime, a country gaining independence, or establishment of new institutions or facilities; groundbreaking achievements in arts or science; beginning of a new era | receiving news that the Berlin Wall has fallen; watching a scene on the opening of the Berlin Wall during a seminar |  | 2 | 1.9 |  | 1 | 1.0 |
| 2322 | negative turning points | elicitors related to negative turning points at a societal, political, or cultural level such as a change of government for the worse, conquest of a country by enemies, or institutions or facilities being closed down; irrecoverable loss of cultural, artistic, or scientific achievements; end of an era | standing in the garden of a deserted building where an advanced training facility had moved out a few days earlier |  | 1 | 0.9 |  | 0 | 0.0 |
| 2331 | social justice/ protection of others/self-sacrifice | elicitors related to social justice such as fighting for equality and against injustice, charity work, protecting others (at one’s own cost), advocating one’s conviction, dying a hero’s death, or protection of animals and nature | giving a talk on the reconciliation of the SED dictatorship; my friend trying to help a homeless woman who had to spend the night outside when the temperature was -10° Celsius |  | 2 | 1.9 |  | 0 | 0.0 |

**S1 Table** *continued.*

|  |  |  |  |  | **Moving personal experience** | |  | **Being-moved prototype^a^** | |
| --- | --- | --- | --- | --- | --- | --- | --- | --- | --- |
| **Number** | **Label** | **Description** | **Examples from study^b^** |  | ***n*** | **%** |  | ***n*** | **%** |
| 2332 | social injustice/ violence against others | elicitors related to social injustice and violence such as discrimination against minorities, war, genocide, terrorism, physical or sexual abuse, cruelty to animals, or destruction of the environment | watching a documentary on the Korean war |  | 1 | 0.9 |  | 0 | 0.0 |
| 2341 | participation in meaning-making system | elicitors related to participation in a meaning-making system or some greater entity such as a political or religious community or a club; feeling close to God, stars, or other positive figures; feeling connected to something higher or larger | feeling that God is talking to me through a friend who knew how I was feeling although I hadn’t told her; Bayern [soccer team] winning the Champions League; while driving my car, I suddenly felt connected to everything around me |  | 3 | 2.8 |  | 1 | 1.0 |
| 2342 | turning away from meaning-making system | elicitors related to turning away from a meaning-making system such as having a crisis of meaning or faith, apostasy, or seeking contact with deviant individuals | - |  | 0 | 0.0 |  | 0 | 0.0 |
| *24 Aesthetics-related elicitors* | | | | | | | | | |
| 2411 | beauty of nature | elicitors related to the beauty of nature | seeing a real iceberg for the first time; seeing “Hierve el Agua” in Mexico; seeing the sun set over the sea after a strenuous three-day hike in Spain |  | 3 | 2.8 |  | 0 | 0.0 |
| 2412 | displeasing nature | elicitors related to aesthetically displeasing nature | - |  | 0 | 0.0 |  | 0 | 0.0 |

**S1 Table** *continued.*

|  |  |  |  |  | **Moving personal experience** | |  | **Being-moved prototype^a^** | |
| --- | --- | --- | --- | --- | --- | --- | --- | --- | --- |
| **Number** | **Label** | **Description** | **Examples from study^b^** |  | ***n*** | **%** |  | ***n*** | **%** |
| 2421 | human-made aesthetics | elicitors related to the beauty of art or design such as music, painting, film, literature, or theatre | watching the dance performance “The Open Square” by the Staatsballett; concert of the Santo Tomas Singers; listening to the choir sing during a rehearsal of Bach’s St. Matthew Passion; singing a song by Debussy with my friends out in the street; watching a dance film; watching the online video of ISS commander Hadfield; seeing the fireworks in Hamburg on New Year’s Eve; visiting a Berlin Christmas market |  | 9 | 8.5 |  | 1 | 1.0 |
| 2422 | human-made displeasing aesthetics | elicitors related to aesthetically displeasing art or design such as music, painting, film, literature, or theatre | - |  | 0 | 0.0 |  | 0 | 0.0 |
| ***3 Respondent’s role in event: Involvement and psychological distance^d^*** | | | | | | | | | |
| *31 Being moved by one’s own action or thought* | | | | | | | | | |
| 311 | no distance from one’s own action | no distance from the self “here and now” is evident in the responses | ^e^ |  | 1 | 0.9 |  | - | - |
| 312 | some distance from one’s own action | responses show some distance from the self “here and now,” for instance, through taking another’s perspective on one’s own action, remembering the past, counterfactual thinking, or anticipating the future | ^e^ |  | 10 | 9.4 |  | - | - |
| *32 Being moved by someone with whom respondent has a personal/close relationship* | | | | | | | | | |
| 321 | active involvement and taking another’s perspective | respondent is moved through interacting with another person; distance through taking another’s perspective | ^e^ |  | 58 | 54.7 |  | - | - |

**S1 Table** *continued.*

|  |  |  |  |  | **Moving personal experience** | |  | **Being-moved prototype^a^** | |
| --- | --- | --- | --- | --- | --- | --- | --- | --- | --- |
| **Number** | **Label** | **Description** | **Examples from study^b^** |  | ***n*** | **%** |  | ***n*** | **%** |
| 322_23 (combination of 322 and 323) | no active involvement | respondent is moved by witnessing something that another person does or experiences at that moment (but does not interact with the other person) or by learning about some past or anticipated future experience of the other person | ^e^ |  | 13 | 12.3 |  | - | - |
| *33 Being moved by someone with whom respondent has no personal relationship* | | | | | | | | | |
| 331 | active involvement and taking another’s perspective | respondent is moved through interacting with another person; distance through taking the other person’s perspective | ^e^ |  | 5 | 4.7 |  | - | - |
| 332_33 (combination of 332 and 333) | no active involvement | respondent is moved by witnessing something that another person does or experiences at that moment (but does not interact with the other person) or by learning about some past or anticipated future experience of the other person | ^e^ |  | 6 | 5.7 |  | - | - |
| *34 Being moved by fiction and aesthetics* | | | | | | | | | |
| 341 | content-related | respondent is moved by the content of a narrative, picture, or song, by what fictitious persons or characters are experiencing | ^e^ |  | 6 | 5.7 |  | - | - |
| 342 | form-related | respondent is moved by music, art, or the beauty of nature per se, by the sound, looks, harmony, or stylistic features | ^e^ |  | 7 | 6.6 |  | - | - |

**S1 Table** *continued.*

|  |  |  |  |  | **Moving personal experience** | |  | **Being-moved prototype^a^** | |
| --- | --- | --- | --- | --- | --- | --- | --- | --- | --- |
| **Number** | **Label** | **Description** | **Examples from study^b^** |  | ***n*** | **%** |  | ***n*** | **%** |
| ***4 Cognitive appraisals^f^*** | | | | | | | | | |
| *41 Relevance of the elicitor* | | | | | | | | | |
| 4111 | familiar/ expected | situation or event is familiar, has been experienced before, or developed as expected | this is normal; as in every year; I have experienced several times; because I was familiar with the situation; I knew my friend would arrive this evening; her death did not come suddenly; I had expected that I would feel moved; I had seen a dead person twice before |  | 12 | 11.3 |  | 3 | 2.9 |
| **4112** | **unfamiliar/ unexpected** | **situation or event is unfamiliar, is experienced for the first time, or developed suddenly and/or unexpectedly** | suddenly; that it came very surprisingly; at first I was very surprised; unexpectedly; never before in my life; happened by chance; that hasn’t happened to me for quite some time; I was not prepared for this; which you consider extraordinary or special; if an experience transports you into a world where you have never been before or into which you rarely advance; you are not used to it |  | **35** | **33.0** |  | **29** | **28.2** |
| **4121** | **personal relevance/ involvement** | **event reminds one of one’s own experiences or of persons whom one knows, is related to or experienced as part of oneself** | recalling my own experiences; against the background of my own losses experienced during my childhood; thinking that my mother was born in this very country only four years after this war; I thought about what it would be like if something like this happened to me; a boy about my age; it made me realize that an exhausting year lies behind me; it usually has to do with your own self; it is important that the situation has personal appeal; when related to your own experiences, fears, or hopes; a situation which is similar to your own; it is important to me |  | **28** | **26.4** |  | **30** | **29.1** |

**S1 Table** *continued.*

|  |  |  |  |  | **Moving personal experience** | |  | **Being-moved prototype^a^** | |
| --- | --- | --- | --- | --- | --- | --- | --- | --- | --- |
| **Number** | **Label** | **Description** | **Examples from study^b^** |  | ***n*** | **%** |  | ***n*** | **%** |
| 4122 | no personal relevance/ involvement | person makes it explicit that the event or stimulus is unrelated to her/him; it is unclear why the person is emotionally affected by the event, as one “should not” be affected | I actually didn’t have anything to do with this; but I didn’t know the deceased |  | 2 | 1.9 |  | 0 | 0.0 |
| 4131 | intrinsically pleasant | eliciting stimulus or situation is described as pleasant or beautiful per se, irrespective of one’s own goals or emotions in the situation; the focus is on the “objective” quality of the stimulus | it was beautiful; the sound was just wonderful; the paper was nicely designed; wonderful composition and performance; the choir was so good; you experience a very nice situation |  | 8 | 7.5 |  | 2 | 1.9 |
| 4132 | intrinsically unpleasant | eliciting stimulus or situation is described as unpleasant or ugly per se, irrespective of one’s own goals or emotions in the situation; the focus is on the “objective” quality of the stimulus | at first I thought that this man was an “old, crazy man”; the music is merciless and frightful |  | 2 | 1.9 |  | 0 | 0.0 |
| *42 Implications* | | | | | | | | | |
| 4211 | causation by self | person sought or created the emotion-eliciting situation or had someone create the situation for her/him; person was actively and willingly involved in how the situation developed | now I have made this dream come true; in which you are involved (in the production); I wanted to talk about and settle this issue |  | 4 | 3.8 |  | 0 | 0.0 |
| 4212 | causation by others/chance | the emotion-eliciting situation happened to the person; the situation was caused by others or chance | an awkward situation turns into my positive fate, it’s like a wonder; told me; when moved, the person does not act herself, something happens to the person that is internally or externally caused |  | 4 | 3.8 |  | 1 | 1.0 |

**S1 Table** *continued.*

|  |  |  |  |  | **Moving personal experience** | |  | **Being-moved prototype^a^** | |
| --- | --- | --- | --- | --- | --- | --- | --- | --- | --- |
| **Number** | **Label** | **Description** | **Examples from study^b^** |  | ***n*** | **%** |  | ***n*** | **%** |
| 4221 | advancement of goals | the moving event improved a person’s situation or lets the person expect a positive turn of events; a negative situation will improve; the person is looking forward to the future | I had the impression that at this moment everything is possible and that a huge opportunity has opened up; I had always wanted to have a cat; therefore I felt certain that he would invest energy into “our thing” and advance it; if an event turns out well; if you experience something you have dreamt about for a long time |  | 9 | 8.5 |  | 3 | 2.9 |
| 4222 | interference with goals | important goals will not be reached as a consequence of the moving event; someone will suffer or have a hard time coping with the situation; the situation will get worse; the future will not be as desired; the person dreads the future | I felt that this loss will trouble me for years to come; I had wanted to visit her that day [but it was not possible because she had died]; something that has raised fears for a long time |  | 3 | 2.8 |  | 1 | 1.0 |
| *43 Coping potential* | | | | | | | | | |
| 4311 | general controllability | the situation shows that, in principle, it is possible to control or cause certain events | - |  | 0 | 0.0 |  | 0 | 0.0 |
| 4312 | general uncontrol­lability | the situation underscores the existential truth that, in principle, certain events cannot be controlled or changed; makes the person think about the general limitations of individual agency or the finitude of everything | every life will be over sometime; how fragile and frail life really is; how quickly time goes by and how transient everything is; I realized that everything is finite; I am, we are all fallible human beings; even though you know precisely that you could not have changed anything; we all overestimate our being and works; it can happen to anyone; you usually experience something about which you feel that it confronts you with the basics of life |  | 15 | 14.2 |  | 4 | 3.9 |
| 4321 | personal control | the person is able to influence the future development of this specific situation or has control over this situation | - |  | 0 | 0.0 |  | 0 | 0.0 |

**S1 Table** *continued.*

|  |  |  |  |  | **Moving personal experience** | |  | **Being-moved prototype^a^** | |
| --- | --- | --- | --- | --- | --- | --- | --- | --- | --- |
| **Number** | **Label** | **Description** | **Examples from study^b^** |  | ***n*** | **%** |  | ***n*** | **%** |
| 4322 | personal uncontrol­lability | the person is not able to control or change this specific situation; the person is overly challenged or has lost control | there also was no opportunity to talk to her any longer; I felt helpless; it was hard that I could only stand by, but not act |  | 3 | 2.8 |  | 0 | 0.0 |
| *44 Normative significance* | | | | | | | | | |
| **4411** | **positive salience of agency** | **agency as an ideal is realized in the situation; fulfilment of or positive deviations from expectations for mastery, control, autonomy, independence, competence, self-esteem, or self-confidence** | which demanded a great deal of me; when I brought it up this time, he expressed his decision clearly and with strong commitment; it’s great that you dare to sing in front of other people; overall it was just impressive how he really wanted to score a success; he managed to get all of this done without me noticing it; spontaneously, voluntarily, and authentically expressed praise; when you have invested a lot of energy into something and now stand in front of the final result and are very satisfied |  | **32** | **30.2** |  | 6 | 5.8 |
| **4412** | **negative salience of agency** | **agency as an ideal is challenged or threatened in the situation; unfulfilled needs for agency; negative deviations from expectations for mastery, control, autonomy, independence, competence, self-esteem, or self-confidence** | being vulnerable; I’m actually not fit enough for an outside left [in soccer]; I didn’t get to do it because she did not want to/could not do it; we had tried helping her without much success; helpless; physical inability; I felt restricted in my freedom of expression; the feeling that I am not able to implement what I was thinking and saying; unfortunately received very little instruction; she lay in bed apathetically and with fast, short breathing; nature as a challenge; this presence is overtaxing; when you watch other people in difficult situations |  | **44** | **41.5** |  | 8 | 7.8 |

**S1 Table** *continued.*

|  |  |  |  |  | **Moving personal experience** | |  | **Being-moved prototype^a^** | |
| --- | --- | --- | --- | --- | --- | --- | --- | --- | --- |
| **Number** | **Label** | **Description** | **Examples from study^b^** |  | ***n*** | **%** |  | ***n*** | **%** |
| **4421_31 (combination of 4421 and 4431)** | **positive salience of connected­ness/ prosociality** | **connectedness/prosociality as an ideal is realized in the situation; fulfilment of or positive deviations from expectations for closeness, love, connection, respect, community spirit, altruism, or prosocial behavior** | it was good to be hugged by someone who shared this moment; a very intimate conversation; that my sister confided this to me; a group of very nice, like-minded friends; made a present for me and gave it to me; that at that moment I had just naturally helped the man; the natural inclination to protect this small life, which set in at once; this [writing a poem for me] must have demanded a lot of time; she just embraced me and prayed for me; unified with nature; you are allowed to share something; when someone else does or says something nice; the exceptional selflessness of another person |  | **74** | **69.8** |  | 13 | 12.6 |
| **4422_32 (combination of 4422 and 4432)** | **negative salience of connected­ness/ prosociality** | **connectedness/prosociality as an ideal is challenged or threatened in the situation; unfulfilled social needs; negative deviations from expectations for closeness, love, connection, respect, community spirit, altruism, or prosocial behavior** | people turned away and tried to avoid eye contact with the others; she withdrew from everyone; very likely we would never again all be able to meet in one place; although no one believed in me; who had been kicked out of the station when it was freezing cold; that she was left alone in this situation; that within four months my second direct supervisor had been fired in the worst way; because I knew that of course I could not help; usually others don’t think I’m right, instead they say I’m wrong; when you recognize injustices |  | **44** | **41.5** |  | 1 | 1.0 |
| 4441 | appropriate expression of emotion | the person perceives her/his emotions and underlying appraisals to be appropriate, conforming to the social norm, or comprehensible | I know that this [losing control of your emotions] is completely ok; nevertheless, I like it better when it [feeling moved] shows |  | 1 | 0.9 |  | 1 | 1.0 |

**S1 Table** *continued.*

|  |  |  |  |  | **Moving personal experience** | |  | **Being-moved prototype^a^** | |
| --- | --- | --- | --- | --- | --- | --- | --- | --- | --- |
| **Number** | **Label** | **Description** | **Examples from study^b^** |  | ***n*** | **%** |  | ***n*** | **%** |
| 4442 | inappropriate expression of emotion | the person explicitly characterizes her/his emotions and underlying appraisals as inappropriate, not conforming to the social norm, exaggerated, or incomprehensible | looking back, I found that my sadness was dispropor-tionate; the moment was so banal… I am almost ashamed to write about it now; other thoughts were: “don’t be so prissy”; which [the emotional reaction] at that moment and also afterwards was/is puzzling to me |  | 4 | 3.8 |  | 1 | 1.0 |
| *45 Complexity* | | | | | | | | | |
| 4511 | simple appraisal | the cognitive appraisal is explicitly characterized as simple and unambiguous; it is clear how the stimulus or situation is to be evaluated | - |  | 0 | 0.0 |  | 0 | 0.0 |
| 4512 | mixed/ complex appraisal | the cognitive appraisal is explicitly characterized as mixed, complex, or complicated | but still; at the same time; even though; many images and thoughts at once; this poignant mixture |  | 7 | 6.6 |  | 5 | 4.9 |
| ***5 Subjective feelings*** | | | | | | | | | |
| *51 “Classical” emotion dimensions* | | | | | | | | | |
| **51011** | **pleasant** | **pleasant, positive, good feelings; feeling well** | a very good feeling; somehow nice; positive feelings; positive events; a positive experience; a deviation from your average mental state toward the positive; it’s nice to feel moved |  | 20 | 18.9 |  | **35** | **34.0** |
| **51012** | **unpleasant** | **unpleasant, negative, bad feelings; feeling ill** | negative feelings; pain; burdensome and almost unbearable; negative events; a deviation from your average mental state toward the negative |  | 8 | 7.5 |  | **26** | **25.2** |
| 51021 | positive low arousal | person is in a state of low arousal that she/he tends to experience as positive; feels relaxed, calm, or at ease | relaxation; very calm; calmed down; all tension fell away; even temper; I was serene; laid-back mood |  | 16 | 15.1 |  | 7 | 6.8 |
| 51022 | negative high arousal | person is in a state of high arousal that she/he tends to experience as negative; feels tense, nervous, agitated, or unsettled | nervousness; uneasy; fairly anxious; I became very agitated; I got hectic; concern is noticeable; deep shock |  | 15 | 14.2 |  | 8 | 7.8 |

**S1 Table** *continued.*

|  |  |  |  |  | **Moving personal experience** | |  | **Being-moved prototype^a^** | |
| --- | --- | --- | --- | --- | --- | --- | --- | --- | --- |
| **Number** | **Label** | **Description** | **Examples from study^b^** |  | ***n*** | **%** |  | ***n*** | **%** |
| 51031 | negative low arousal | person is in a state of low arousal that she/he tends to experience as negative; feels tired, weak, or listless | completely weak; tiredness; feeling of exhaustion; I felt dead tired; droopy and exceptionally sluggish; you would like to spend the days in bed |  | 7 | 6.6 |  | 1 | 1.0 |
| 51032 | positive high arousal | person is in a state of high arousal that she/he tends to experience as positive; feels excited, motivated, or full of energy | very excited; felt alert; alive; very lively; exuberance; intent in a positive way and focused; you are excited per se; excitement |  | 20 | 18.9 |  | 8 | 7.8 |
| 51041 | potency/ greatness | feelings accompanying a high position within a hierarchy, feelings of power, status, and superiority, but also feelings of greatness and strength | I felt very strong, mentally; big; powerful |  | 2 | 1.9 |  | 1 | 1.0 |
| 51042 | humility/ smallness | feelings accompanying a low position within a hierarchy; feelings of humility and inferiority, but also feelings of smallness, weakness, or powerlessness | humble; humility; immense powerlessness; a feeling of helplessness; I felt so very small; a daunting feeling arose in me; in the case of great suffering, the person feels helpless |  | 8 | 7.5 |  | 4 | 3.9 |
| 51051 | intense experience | the emotion or the entire situation is experienced very intensely or consciously; person is immersed completely in the “here and now” | you are filled with emotion; I was completely in the moment; everything was closer; at that moment everything else was forgotten; you lose any sense of time and feel as if in another world; here and now but yet somewhere completely different; there are no other thoughts or feelings at that moment |  | 20 | 18.9 |  | 25 | 24.3 |
| 51052 | shallow experience | the emotional experience in the situation was generally of low intensity, flat; the person hardly felt any emotion, was unaffected | everything was further removed |  | 1 | 0.9 |  | 0 | 0.0 |
| *52 Other emotion dimensions* | | | | | | | | | |
| 52011 | diverting attention | feelings of boredom or monotony; attention is diverted from an uninteresting stimulus | - |  | 0 | 0.0 |  | 0 | 0.0 |

**S1 Table** *continued.*

|  |  |  |  |  | **Moving personal experience** | |  | **Being-moved prototype^a^** | |
| --- | --- | --- | --- | --- | --- | --- | --- | --- | --- |
| **Number** | **Label** | **Description** | **Examples from study^b^** |  | ***n*** | **%** |  | ***n*** | **%** |
| 52012 | paying attention | person feels surprise, amazement, astonishment, interest, curiosity, fascination or is captivated or spellbound; all emotions that cause attention to be focused on a stimulus | startled; receptive; curiosity; amazement; surprise; observant; I was perplexed; completely focused on the moving element; attention, listening or watching closely; I sat at the edge of my seat; my thoughts and feelings are directed at a concrete event; I wanted to sit down to absorb more sensations; being moved requires complete concentration |  | 23 | 21.7 |  | 18 | 17.5 |
| 52021 | controll­ability/ smallness of emotion | feeling or the emotional impact of a stimulus is easy to control and suppress; the feeling is small, insignificant | - |  | 0 | 0.0 |  | 0 | 0.0 |
| **52022** | **uncontrol-lability/ magnitude of emotion** | **feeling is uncontrollable, strong, deep, wide, overwhelming, sudden, intrusive, or cannot be regulated; person’s body is too narrow for the emotion** | strong emotion; a very deep feeling, pervasive; I felt as if a giant wave carried me away, drowning in feelings; I was completely carried away; it very much overburdened me emotionally; without my being able to regulate them [the tears]; you are overwhelmed; an overflowing well of emotion; a moment that is so powerful; the experience engrosses your whole body and mind; being moved is characterized by your inability to regulate or influence this emotion; loss of control; the person is not able to really handle her feelings; evokes extreme emotions, which can be of any kind; being moved means to be exposed to an inner force |  | **32** | **30.2** |  | **53** | **51.5** |
| 52031 | resisting | feeling that one has to resist something or pull oneself together; person does not want to allow the emotion, wants to suppress it | I tried to work against it and stay calm so that I did not lose my composure; that I tried to conceal the emotions from the persons sitting next to me; I had to suppress [the tears]; I didn’t want to drown in erroneous hope; possibly particularly intense emotions are being covered… — poker face; others try to suppress the emotion |  | 7 | 6.6 |  | 3 | 2.9 |

**S1 Table** *continued.*

|  |  |  |  |  | **Moving personal experience** | |  | **Being-moved prototype^a^** | |
| --- | --- | --- | --- | --- | --- | --- | --- | --- | --- |
| **Number** | **Label** | **Description** | **Examples from study^b^** |  | ***n*** | **%** |  | ***n*** | **%** |
| 52032 | letting go | person lets go and does not try to hide or control the emotion; the situation is accepted, the person gives in to it; there is no need for pretense | “melting into” your feeling; you let many things out which you would not do otherwise; being able to give free rein to your feelings; if you give yourself over to the feeling; there are people who accept and let out the emotion |  | 6 | 5.7 |  | 9 | 8.7 |
| 52041 | turning away from the outside world | person is lost in thought, hardly notices his/her surroundings, turns away from the outside world, focuses on his/her own experience and thoughts in the situation, and forgets everything around him/her | I got very pensive; it’s like being in an empty room: you hear nothing, don’t see what’s going on around yourself; at that moment I cannot think of anything; I was not approachable for several minutes; you don’t want to deal with the outside world; the person veers away from reality in her thoughts; everything else seems remote and unimportant |  | 10 | 9.4 |  | 11 | 10.7 |
| 52042 | opening up to the outside world | the feeling opens the person to someone or something else; the person perceives his/her surroundings more consciously and is responsive and open to new impressions and experiences | all sorrows and thoughts, problems, and even my ego were being completely turned off; you can be touched and are open to the currents of the world; I am more vulnerable, because my “soft” side is stimulated; being open to others and to yourself; you take in everything around yourself, absorb it like a sponge |  | 2 | 1.9 |  | 9 | 8.7 |
| 52051 | lightness | person feels light, unburdened, floating, detached | I feel light and untroubled; I feel light inside; you fly or hover; like a dream! everything feels light!; weightless; floating, liberated from the pull of gravity |  | 11 | 10.4 |  | 3 | 2.9 |
| 52052 | gravity | person feels heavy, as if a heavy weight rests on him/her; something pulls him/her down | heavy; heavy feeling on the chest; bodily feeling of heaviness |  | 4 | 3.8 |  | 0 | 0.0 |

**S1 Table** *continued.*

|  |  |  |  |  | **Moving personal experience** | |  | **Being-moved prototype^a^** | |
| --- | --- | --- | --- | --- | --- | --- | --- | --- | --- |
| **Number** | **Label** | **Description** | **Examples from study^b^** |  | ***n*** | **%** |  | ***n*** | **%** |
| 52061 | extraordinary/ divine | feelings evoked through contact with something higher, divine, or supernatural; person feels connected to what is good, worthy, or moral and feels awe or (moral) elevation; feelings of pureness, magic, brightness | it was almost magical, this moment; I had an elevating feeling; sublime; I had the feeling that I had witnessed something very big; a perfect moment; “divine”; that God can talk to me in this way; when you suddenly feel something that is not ordinary; you experience something special |  | 9 | 8.5 |  | 10 | 9.7 |
| 52062 | ordinary/ profane | feelings associated with everyday life; the experience is ordinary, banal, or trivial | - |  | 0 | 0.0 |  | 0 | 0.0 |
| *53 Specific emotions* | | | | | | | | | |
| **53011** | **joy** | **feelings of joy, happiness, satisfaction, or cheerfulness** | I am happy; happiness; the intense feeling of happiness; made me happy; deep joy; overjoyed; cheerful; bliss; elation; euphoria; I was pleased; satisfied; an experience after which you are full of happiness |  | **52** | **49.1** |  | **32** | **31.1** |
| 53012 | disappoint-ment | feelings of disappointment, disillusionment, disenchantment | which very much disappointed me; deep disappointment |  | 1 | 0.9 |  | 1 | 1.0 |
| 53021 | solace | feelings of solace, of being consoled or given comfort | consoles you |  | 1 | 0.9 |  | 0 | 0.0 |
| **53022** | **sadness** | **feeling sad, depressed, downhearted, sorrowful, or down** | I was sad; feeling blue; sadness; unhappy; great wistfulness; grief; it hurt me a lot; feeling of loss; it depressed me very much; melancholy; tragic; it can be a deeply sad moment; the feeling is very close to sadness |  | **36** | **34.0** |  | **28** | **27.2** |

**S1 Table** *continued.*

|  |  |  |  |  | **Moving personal experience** | |  | **Being-moved prototype^a^** | |
| --- | --- | --- | --- | --- | --- | --- | --- | --- | --- |
| **Number** | **Label** | **Description** | **Examples from study^b^** |  | ***n*** | **%** |  | ***n*** | **%** |
| **53031** | **relatedness/ empathy/ appreciation** | **feelings of affection, closeness, tenderness, sympathy, love, empathy, compassion, admiration, or gratitude** | empathic; a feeling of belonging; relatedness; gratitude; thankful; we fell in love; love; admiration; compassion/ mercifulness; I felt with the relatives; being happy for others; I like people; my heart feels very big; I’m happy that he feels the same; you feel secure; you feel a deep emotional connection with another person; characterized by a strong connection with another person; when you develop strong feelings for someone or something |  | **53** | **50.0** |  | 24 | 23.3 |
| 53032 | depreciation/ hatred/cold-heartedness | ill feelings towards others such as refusal, antipathy, contempt, or hatred; person is unable or unwilling to empathize with others | hatred; me against the rest of the world, because the rest don’t understand what this is all about for me anyway and why I have these strong emotions |  | 1 | 0.9 |  | 1 | 1.0 |
| 53041 | insight | person suddenly has an insight, a flash of inspiration, or an “aha” moment; suddenly something makes sense; the person understands at last | the feeling when you suddenly understand; inspiration; it was like an “aha” experience; it was a feeling of realization; a moment of insight; the moment when I became aware that…; when I realized…; you are moved when you have had an insight about something |  | 7 | 6.6 |  | 3 | 2.9 |
| 53042 | confusion | person feels confused, shocked or disbelieving or cannot understand; things do not make sense | somewhat confused; disbelief; incomprehension; discomposure; this cannot be true; feeling of being frightened; a little shock; some sort of “freezing”; shocked |  | 11 | 10.4 |  | 4 | 3.9 |
| 53051 | relief | person feels relieved, secure, and calmed down after having been upset | relief; relieved; it took a great load off my mind; it had something liberating |  | 12 | 11.3 |  | 0 | 0.0 |
| 53052 | anxiety | person feels anxious, uneasy, nervous, troubled or worried or is afraid of something | I was anxious; aggrieved; a little afraid; scary; oppressive feeling; incessant trepidation; worry; a very strong anxiety; fear of the future; uncertainty |  | 14 | 13.2 |  | 5 | 4.9 |

**S1 Table** *continued.*

|  |  |  |  |  | **Moving personal experience** | |  | **Being-moved prototype^a^** | |
| --- | --- | --- | --- | --- | --- | --- | --- | --- | --- |
| **Number** | **Label** | **Description** | **Examples from study^b^** |  | ***n*** | **%** |  | ***n*** | **%** |
| 53061 | pride/feeling appreciated | feeling proud of oneself, esteemed by others, or honored; feeling that one is a valuable person or is liked; feelings of self-esteem | pride; I was a little proud; I also felt a little honored; I’m appreciated; he made me feel that he loves and respects me; I’m a good person; felt liked, almost loved; I felt that the affection and love I have for my nephew is being reciprocated; that you feel important |  | 14 | 13.2 |  | 3 | 2.9 |
| 53062 | shame/feeling disrespected | feeling ashamed, embarrassed, guilty, or remorse; having a bad conscience; feeling one has done something wrong; feeling worthless, underappreciated, or disrespected | shame; ashamed; embarrassment; I felt guilty; many people find it embarrassing to be moved |  | 4 | 3.8 |  | 1 | 1.0 |
| 53071 | hope | feelings of hope, confidence, and optimism | hope; I felt encouraged; fierce courage; courage to face life; confident that everything in life will somehow fall into place; a moved person thinks that right now nothing at all can happen to her/him |  | 7 | 6.6 |  | 3 | 2.9 |
| 53072 | hopelessness/ despair | feelings of hopelessness, despair, despondence, or pessimism; feeling empty or hollow | despair; feeling of helplessness and hopelessness; I was desperate; everything felt meaningless; nothing felt important any longer; I have never before felt this empty; my heart felt hollow; a kind of implosion |  | 9 | 8.5 |  | 2 | 1.9 |
| **53081** | **intuition** | **precedence of intuitive feeling over rational thinking; person acts spontaneously or even irrationally; person loses a sense of reality; feelings of dreaminess, longing, or nostalgia** | feeling and thinking: intuitive; acts emotionally, not rationally; acts irrationally; you don’t think on a logical level any longer; actions can be impulsive; comparable to a state of trance; it took about one hour before I could again discuss in a heard-headed manner; a moving moment is not a moment of thinking but of feeling; which I associate with intense longing |  | 5 | 4.7 |  | **28** | **27.2** |
| 53082 | rationality | precedence of clear thinking over intuition; keeping a clear head; perceiving the situation realistically | I rationalized the feeling; down-to-earth; doesn’t act precipitately |  | 2 | 1.9 |  | 1 | 1.0 |

**S1 Table** *continued.*

|  |  |  |  |  | **Moving personal experience** | |  | **Being-moved prototype^a^** | |
| --- | --- | --- | --- | --- | --- | --- | --- | --- | --- |
| **Number** | **Label** | **Description** | **Examples from study^b^** |  | ***n*** | **%** |  | ***n*** | **%** |
| 53091 | placidness/ forgiveness | feelings of being appeased, conciliated, pacified; feeling placid, benign, mild, or soft | I felt mellow; I felt totally soft; it feels soft; when the whole nuisance, negative thoughts suddenly disappear |  | 3 | 2.8 |  | 3 | 2.9 |
| 53092 | anger | feelings of anger, fury, rage, or testiness; being irritated or unnerved | anger; rage; angry; I was mad; somewhat annoying; which bothered me; fits of rage; anger, aggressiveness |  | 8 | 7.5 |  | 14 | 13.6 |
| 53101 | humor | person finds it funny or amusing; person is made to laugh | we both took it with humor; there was some humor in… |  | 2 | 1.9 |  | 0 | 0.0 |
| 53102 | earnestness | person is earnest and not in the mood for joking | - |  | 0 | 0.0 |  | 0 | 0.0 |
| *54 Complexity* | | | | | | | | | |
| 54011 | simple emotion | feeling is characterized as simple, plain, or ordinary | - |  | 0 | 0.0 |  | 0 | 0.0 |
| **54012** | **mixed/ complex emotion** | **feeling is characterized as mixed, complex, multi-layered, or complicated** | at the same time; at the same moment; simultaneously; but also; though still; nonetheless; a lot of emotions simultaneously, overlapping; it was a mixture of many emotions; many emotions which hit you at the same time; a mixture; because it was many feelings mixed |  | **41** | **38.7** |  | 11 | 10.7 |
| ***6 Bodily symptoms^g^*** | | | | | | | | | |
| **6011** | **warmth** | **bodily sensations of warmth; warmth in the chest; warm heart** | felt warmth; my chest felt warm; I’m getting warmer; I felt very warm; warmth surging up; inner feeling of warmth; heat shower; hot; warmth flowing through yourself; it feels warm; my heart warmed; warm heart |  | **30** | **28.8** |  | 6 | 5.8 |
| 6012 | coldness | bodily sensations of coldness; person is cold, shivering, freezing | cold; cold feeling; cold hands; my hands got cold |  | 4 | 3.8 |  | 0 | 0.0 |
| 6021 | sweating | person is perspiring, breaks out in a sweat, or has moist hands/a moist back | slight sweating; I sweated; cold sweating; moist hands |  | 6 | 5.8 |  | 0 | 0.0 |
| 6022 | goosebumps/ chills | piloerection; person is getting goosebumps; chills, thrills, shivers down the spine | I’m getting goosebumps; shivers down the spine; a comforting chill (gooseflesh); warm chills; got goosebumps on the arms; the hair on my arms stood up |  | 10 | 9.6 |  | 3 | 2.9 |

**S1 Table** *continued.*

|  |  |  |  |  | **Moving personal experience** | |  | **Being-moved prototype^a^** | |
| --- | --- | --- | --- | --- | --- | --- | --- | --- | --- |
| **Number** | **Label** | **Description** | **Examples from study^b^** |  | ***n*** | **%** |  | ***n*** | **%** |
| **6031** | **tears** | **crying or feeling like crying; tears welling up; moist eyes** | tears; I cried; crying; tears welled up in my eyes; I could have cried; I inevitably have to start crying; maybe I might have been moved to tears; tears of joy; water in the eyes; you cry, for instance |  | **57** | **54.8** |  | 24 | 23.3 |
| 6041 | lump in throat/ choked up | person feels a lump in the throat; throat is choked up and feels tight or obstructed; person is hardly able or unable to speak; speech is impaired | I had a huge lump in the throat; like a lump in the throat; the throat feels tight; could continue speaking only with great effort; unable to speak; I could say nothing; moment of speechlessness; at first you are completely at a loss for words |  | 11 | 10.6 |  | 8 | 7.8 |
| 6051 | salivation/ swallowing | person has to swallow, lots of saliva in the mouth, drooling | I had to swallow; frequent swallowing |  | 2 | 1.9 |  | 0 | 0.0 |
| 6052 | dry mouth/ rarely swallowing | person has a dry mouth, wants to drink, lacks saliva, or swallows rarely | a dry mouth |  | 2 | 1.9 |  | 0 | 0.0 |
| 6061 | increased heart rate | heart beats faster, palpitation of the heart, fast pulse | my heart beat faster; increased heartbeat; throbbing of the heart; racing heart; pulse beat faster |  | 13 | 12.5 |  | 5 | 4.9 |
| 6062 | reduced heart rate | heart beats slower, slow pulse | - |  | 0 | 0.0 |  | 0 | 0.0 |
| 6071 | tingling/ pulsing sensation | tingling, pulsing, pounding, or throbbing feelings in the entire body, limbs, or body parts | at first it prickles; prickling in the stomach and legs; my fingers were tingly; it somehow pulses inside me; “butterflies in the stomach”; almost like a pleasant tingling |  | 12 | 11.5 |  | 2 | 1.9 |
| 6072 | numbness | the whole body or limbs feel numb | somewhat numb |  | 1 | 1.0 |  | 0 | 0.0 |
| 6081 | fast breathing | faster, shallower breathing; being out of breath, panting, or gasping for air | breathed faster; shallow breathing; increased respiratory rate; heavy breathing; as if your breath is taken away |  | 6 | 5.8 |  | 2 | 1.9 |
| 6082 | slow breathing | slower, deeper, more intense breathing; taking a deep breath | deep but calm respiration; drawing a deep breath; deep exhalation |  | 3 | 2.9 |  | 1 | 1.0 |

**S1 Table** *continued.*

|  |  |  |  |  | **Moving personal experience** | |  | **Being-moved prototype^a^** | |
| --- | --- | --- | --- | --- | --- | --- | --- | --- | --- |
| **Number** | **Label** | **Description** | **Examples from study^b^** |  | ***n*** | **%** |  | ***n*** | **%** |
| 6091 | muscle tension | muscles tense, constrict, or cramp; body is tense and uptight; muscles are trembling; physically resisting pressure or some constricting force | tense; tension (i.e., tension of the muscles); sometimes I tense up; something tight in the chest; pressure on the chest; as if something in my chest contracts; the feeling in my heart “constricts”; it felt constricting; was shaky; legs were shaking; slight trembling; being moved is a state during which some sort of tension is spreading across the front of the stomach or chest |  | 22 | 21.2 |  | 5 | 4.9 |
| 6092 | muscle relaxation | muscles relax; physical tension is eased; body relaxes, softens; something in the body expands | relaxed bodily feeling; all body parts become softer; a certain relaxation sets in; relaxation rather than tension, ease |  | 5 | 4.8 |  | 1 | 1.0 |
| 6101 | physical energy | person has much bodily strength and energy; urge to release pent-up physical energy | I felt very strong, physically; strength; the person jumps around; makes (hyper)active; I am more agile or, more precisely, I feel like I could tear out trees; pent-up energy; need for movement; even a caper; adrenalin in high doses |  | 10 | 9.6 |  | 4 | 3.9 |
| 6102 | physical fatigue | bodily weakness and fatigue; person has no strength in the limbs, needs to sit down, is unable to move, or feels paralyzed | bodily fatigue; I was extremely tired; I was physically weak and exhausted; physically paralyzing; faintness of the legs; my eyes were heavy; I was very tired and wanted to sleep |  | 13 | 12.5 |  | 3 | 2.9 |
| 6111 | blushing | person blushes; face turns red; cheeks feel hot | I realized that I turned red; facial rash |  | 2 | 1.9 |  | 1 | 1.0 |
| 6112 | turning pale | person turns pale; face turns white | - |  | 0 | 0.0 |  | 0 | 0.0 |
| 6121 | physical ill-being | person feels physically unwell or sick; nausea or dizziness; feeling like or actually fainting; upset stomach or loss of appetite | dizziness; feeling queasy; sickness; my stomach hurt; heart and stomach hurt; loss of appetite; lump in the stomach; drop in blood pressure |  | 7 | 6.7 |  | 3 | 2.9 |
| 6122 | physical well-being | person feels physically well, healthy | it was a good bodily feeling; I felt good in my body |  | 3 | 2.9 |  | 0 | 0.0 |

**S1 Table** *continued.*

|  |  |  |  |  | **Moving personal experience** | |  | **Being-moved prototype^a^** | |
| --- | --- | --- | --- | --- | --- | --- | --- | --- | --- |
| **Number** | **Label** | **Description** | **Examples from study^b^** |  | ***n*** | **%** |  | ***n*** | **%** |
| 6131 | smiling | person smiles, laughs, or grins | I smiled; smiling; I smiled at him; laughing; I had to grin; a smile that makes the eyes shine bright; cheers; maybe a smile; you laugh |  | 24 | 23.1 |  | 10 | 9.7 |
| 6132 | corners of the mouth down | persons turns down the corners of the mouth | - |  | 0 | 0.0 |  | 0 | 0.0 |
| 6141 | frowning | person frowns; raises or pulls the eyebrows together | furrowed brows |  | 1 | 1.0 |  | 0 | 0.0 |
| 6151 | strong physical response | person speaks of a strong physical response without characterizing it further | elicits bodily reactions; something just happens in the body; physically agitated; that the feeling is not just mentally but physically experienced |  | 1 | 1.0 |  | 3 | 2.9 |
| 6152 | no/little physical response | person explicitly says that there was no physical response; person did not feel or recognize any physical changes | I had no bodily reactions at that moment or maybe I did not perceive them; [the feeling] physically was not very strong; in this special case there were no actual bodily sensations to be felt |  | 3 | 2.9 |  | 0 | 0.0 |
| ***7 Cognitive consequences^h^*** | | | | | | | | | |
| 711 | strong influence | person says that the experience had a strong, important, or enduring influence on his/her thoughts, life, or self-concept without further characterizing this influence | you are a different person; can influence my future decisions; it flowed through the entire day, I was more attentive, more in the moment; which sets something in motion inside yourself; can bring about big changes in a person’s life; can make you think, feel differently |  | 4 | 3.8 |  | 7 | 6.8 |
| 712 | no/weak influence | person says that the experience had no or only a very brief influence on his/her thoughts, life, or self-concept | none; it did not affect my further thoughts or actions; it is not like “being moved” had any lasting impact; this does not have a noticeable influence on my everyday life |  | 4 | 3.8 |  | 0 | 0.0 |

**S1 Table** *continued.*

|  |  |  |  |  | **Moving personal experience** | |  | **Being-moved prototype^a^** | |
| --- | --- | --- | --- | --- | --- | --- | --- | --- | --- |
| **Number** | **Label** | **Description** | **Examples from study^b^** |  | ***n*** | **%** |  | ***n*** | **%** |
| **721** | **keep thinking** | **moving experience or emotion is vividly or easily recalled; the person keeps thinking about and will not forget the experience** | a lot of thinking afterwards; this feeling made me think of its elicitors for hours to come; I won’t forget it so soon; in every free, dreamy minute I remember this situation; I just try to preserve this moment for me, to not let it fade away; my girlfriend and I tried to stay in the images even longer, to reflect; I paused and briefly looked pensively into the air; I wondered what made me so sad that I needed comfort; events that make you pause and cause thinking and pondering; makes you think |  | **35** | **33.3** |  | 13 | 12.6 |
| 722 | stop thinking | moving experience or emotion is hard to recall; person does not like to recall it, wants to stop thinking about it, and would rather forget it | I tried to distract myself from it; distraction from the situation |  | 2 | 1.9 |  | 0 | 0.0 |
| **731** | **finding meaning** | **moving experience made person realize or guess some truth, which is not characterized any further; person gained some insight or discovered what is important or meaningful** | it focuses on what counts in life; the hike gained sense and meaning as I experienced that moment; don’t wait, don’t postpone really important things, life does not wait; it can make you reflect on your entire previous life; something like a truth capsule, which I occasionally enter to remind myself of the essential; when you can suddenly face crude facts and feel your own strengths/weaknesses; everything will turn out well, in the end; never again will anything else be more important or meaningful; previously decided things usually become secondary or even obsolete; you become aware of things that otherwise lie rather hidden |  | **27** | **25.7** |  | 21 | 20.4 |

**S1 Table** *continued.*

|  |  |  |  |  | **Moving personal experience** | |  | **Being-moved prototype^a^** | |
| --- | --- | --- | --- | --- | --- | --- | --- | --- | --- |
| **Number** | **Label** | **Description** | **Examples from study^b^** |  | ***n*** | **%** |  | ***n*** | **%** |
| 732 | sense of meaningless­ness | moving experience made person realize the meaninglessness and insignificance of the experience or his/ her entire life; things have lost their meaning | I didn’t want to live on |  | 1 | 1.0 |  | 0 | 0.0 |
| 741 | recognize value of agency | person realized the importance of values and goals related to agency, autonomy, competence, or self-reliance/self-esteem; importance of personal freedom, freedom of opinion, physical integrity, personal security, or dignity | how important the freedom of a person is, the greatest good of a person; not to accept everything as a given; made me aware that I live in a secure environment offering chances and possibilities, which you should pay much more attention to instead of accepting everything as it happens; it was crystal clear to me that you have to move in life; how wonderful it is when some can save lives with their hands |  | 8 | 7.6 |  | 1 | 1.0 |
| 742 | recognize agency at others’ costs as worthless | person realized that agency at others’ costs and egoistic, selfish, reckless, boastful, or unsocial behavior are despicable | I feel that my father is a giant egoist |  | 1 | 1.0 |  | 0 | 0.0 |
| 751_61 (combination of 751 and 761) | recognize value of connectedness/ prosociality | person realized the importance of specific relationships or connectedness in general, of loving and being loved, caring for others, being prosocial, and advocating for collective values and norms | he showed me: not everyone does this [help], yet it is so important; I became aware of how important he was to me; maybe that is the crucial point, when others participate; that my claim for a respectful and fair work environment is not a weakness and not “do-goodism” but a genuine necessity; you have to settle important things with those who are important to you while you still can; you should appreciate your fellow human beings (and favorite stars) as long as they are there for you or, rather, produce music; intimacy and community, togetherness and shared happiness—that’s not the worst; I was impressed by my friend’s good will |  | 24 | 22.9 |  | 3 | 2.9 |

**S1 Table** *continued.*

|  |  |  |  |  | **Moving personal experience** | |  | **Being-moved prototype^a^** | |
| --- | --- | --- | --- | --- | --- | --- | --- | --- | --- |
| **Number** | **Label** | **Description** | **Examples from study^b^** |  | ***n*** | **%** |  | ***n*** | **%** |
| 752_62 (combination of 752 and 762) | recognize connectedness/ prosociality at one’s own disadvantage as worthless | person realized that it is important not to want to belong or to help and be there for others at any cost, or that some relationships are not worthy of being maintained; importance of not depending on others’ love or respect; some people do not deserve help | - |  | 0 | 0.0 |  | 0 | 0.0 |
| 771 | personal improvement | viewing oneself more positively, feeling better, or having higher self-esteem as a consequence of the moving event; person has grown, developed, or made a positive change and became a better person | afterwards I am refined, purified; which made me better myself to some extent; then I would be a better person; everyday life seems easier now; it gave me self-confidence to believe in myself; my behavior got wiser; I became stronger through this intense phase of grief; through this experience I have become considerably more lenient toward myself; after the situation I am calmer, more relaxed, more balanced; because I revised my opinion of the student; the experience helped me continue with new zest for action |  | 21 | 20.0 |  | 5 | 4.9 |
| 772 | personal worsening | viewing oneself more negatively, feeling worse, or having lower self-esteem as a consequence of the moving event; person is disappointed in him-/herself and became a worse person | later on I became depressive, felt somehow guilty; a strong feeling of displacement |  | 2 | 1.9 |  | 0 | 0.0 |

**S1 Table** *continued.*

|  |  |  |  |  | **Moving personal experience** | |  | **Being-moved prototype^a^** | |
| --- | --- | --- | --- | --- | --- | --- | --- | --- | --- |
| **Number** | **Label** | **Description** | **Examples from study^b^** |  | ***n*** | **%** |  | ***n*** | **%** |
| 781 | improvement of relationships | a specific relationship has improved as a consequence of the moving event; person feels closer and more related to others or an entire community | I feel closer to those who have experienced it with me; I later had the feeling of being closely connected with the man in some way; it strongly changed my relationship with my father; I became aware that I want to stay with my girlfriend for the rest of my life; the love for my child and girlfriend intensified even more at that moment; I feel that I can place more trust in my son’s abilities; I feel closer to people when I am moved |  | 8 | 7.6 |  | 1 | 1.0 |
| 782 | worsening of relationships | a specific relationship has gotten worse as a consequence of the moving event; person feels less close or less related to others or an entire community | a family was—pathetically speaking—doomed to fail; I would not even be able to kiss my partner |  | 2 | 1.9 |  | 0 | 0.0 |
| ***8 Action tendencies^i, j^*** | | | | | | | | | |
| 811 | strong influence | person says that the experience had a strong, important, or enduring influence on his/her actions or action tendencies without further characterizing this influence | has contributed to reconsidering my own actions in certain situations; has a profound effect; get active: “roll up your sleeves”; being moved will trigger actions that might not have happened otherwise; have the effect that the person changes his or her future behavior in order to implement what has moved him or her in his or her own life; it’s possible that you might overreact |  | 1 | 1.0 |  | 6 | 5.8 |
| 812 | no/weak influence | person says that the experience had no or very little influence on his/her actions or action tendencies; person denies that being moved promotes any action tendency | it’s not so much something that would change my actions in concrete terms; my subsequent actions were not influenced; my further actions were not affected by it; however, I cannot tell to what extent this influenced my actions, or rather, I did not perceive any change; I do not think that the video affected my subsequent actions; in the wake of the moving moment, actions can change, but they don’t have to; it’s possible that you don’t do anything |  | 10 | 9.7 |  | 3 | 2.9 |

**S1 Table** *continued.*

|  |  |  |  |  | **Moving personal experience** | |  | **Being-moved prototype^a^** | |
| --- | --- | --- | --- | --- | --- | --- | --- | --- | --- |
| **Number** | **Label** | **Description** | **Examples from study^b^** |  | ***n*** | **%** |  | ***n*** | **%** |
| 821 | seeking experience | person plans to do or does something (other than recalling it) to repeat or prolong the moving experience; person wants to re-experience the stimulus or the emotion | I wanted this feeling to recur; I want to have this feeling over and over again; rather keep on watching forever; I looked at it again at a later time; I wanted to watch the video again; I’m determined to attend similar performances more often in the future; I would have liked to have had more moments like this in my life and I hope that some are yet to come; afterwards you long for this moment |  | 12 | 11.7 |  | 3 | 2.9 |
| 822 | avoiding experience | person plans to do or does something to prevent the moving experience from repeating; person wants to leave or end the situation and does not want to re-experience the stimulus or the emotion | I cannot or, rather, do not like to bear this feeling; I went outside and walked to escape the feeling of external pressure; sometimes you want to get out of the situation |  | 2 | 1.9 |  | 1 | 1.0 |
| 831 | sharing experience | person wants to tell or has told others about the moving experience and wants others to share the emotion; person recommends the moving stimulus to others | I told everyone about it; at home, I told my boyfriend right away about this; I shared it on Facebook; I will recommend the film to friends/couples; it’s a pity I can’t really share that with anyone; talk to close others; had the need to pass on that feeling; it is nice when you can pass it on to others; the need to convey the feeling; make your feelings known to the entire world; extremely communicative |  | 9 | 8.7 |  | 9 | 8.7 |
| 832 | keeping quiet about experience | person does not want others to learn of the moving experience or the emotion; being moved is a private emotion | it is an experience that you like to keep to yourself and to enjoy in silence; in no event openly talk about it at once; … falling silent |  | 0 | 0.0 |  | 3 | 2.9 |

**S1 Table** *continued.*

|  |  |  |  |  | **Moving personal experience** | |  | **Being-moved prototype^a^** | |
| --- | --- | --- | --- | --- | --- | --- | --- | --- | --- |
| **Number** | **Label** | **Description** | **Examples from study^b^** |  | ***n*** | **%** |  | ***n*** | **%** |
| 841 | striving for agency | person wants or strives to increase or express his/her agency, competence, capability, or self-esteem; person wants to be (more) self-determined or assertive, to accomplish something, or rewards him-/herself | I was highly motivated afterwards; getting prepared so that you don’t fall into a hole of uncertainty; because I want to change the situation; as a result, I made an intense effort to find a new job; I got up and danced out my emotion; plans to do a lot of things better; you might want to start the next big, promising project right away; it can make you want to change a detail in your attitude or in the person you are |  | 8 | 7.8 |  | 2 | 1.9 |
| 842 | reduction of agency | person wants or strives to reduce or hide his/her agency, competence, or self-esteem; alternatively, person wants to undermine the agency, competence, or self-esteem of others | - |  | 0 | 0.0 |  | 0 | 0.0 |
| 851_61 (combination of 851 and 861) | striving for connectedness/ prosociality | person wants or strives to begin, maintain, or improve relationships, seek social connection, show appreciation of, care for, and help others, and stand up for social values | I called a close person to tell her how important she is to me; I will take even more care with my parents and always be there for them; I have more respect for him; I decided to thank him the very next day and actually did it, too; I will visit her once again in the next few weeks; kudos to my child; I just long for leaning on, holding the person I love; I try to be reliable and helpful; in the long term, it made me get involved with youth exchanges; the wish that it could be like that again [like the great time we had during the beginning of our friendship]; motivates increased social exchange with friends; you can embrace the whole world; you develop a feeling of caring; you may feel the urge to help; acting altruistically in the service of a good cause; a kind of longing to go to the other and to do something good for him as compensation |  | 24 | 23.3 |  | 13 | 12.6 |

**S1 Table** *continued.*

|  |  |  |  |  | **Moving personal experience** | |  | **Being-moved prototype^a^** | |
| --- | --- | --- | --- | --- | --- | --- | --- | --- | --- |
| **Number** | **Label** | **Description** | **Examples from study^b^** |  | ***n*** | **%** |  | ***n*** | **%** |
| 852_62 (combination of 852 and 862) | reduction of connectedness/ prosociality | person wants or strives to reduce contact, break up relationships, distance him-/herself from others, show others that he/she does not like them, stop caring for others, or ignore social values | I was wondering if I should just leave my parents alone for a few weeks or months and have as little contact as possible; motivates social withdrawal; you will be more likely to withdraw |  | 1 | 1.0 |  | 2 | 1.9 |
| ***Additional combination: Consequence for thought and/or action*** | | | | | | | | | |
| 741_841 | recognize value of/strive for agency | see 741 and 841 | see 741 and 841 |  | 15 | 14.3 |  | 3 | 2.9 |
| **751_61_851_61 (combination of 751_61 and 851_61)** | **recognize value of/ strive for connected­ness/ prosociality** | **see 751_61 and 851_61** | **see 751_61 and 851_61** |  | **41** | **39.0** |  | 15 | 14.6 |
| ***9 Concept of being moved*** | | | | | | | | | |
| 91 | distinct emotion | person has a specific understanding of being moved as a distinct emotional state | ^e^ |  | 106 | 100.0 |  | 103 | 100.0 |
| 92 | any emotion  or moving physically | person understands being moved as having any emotion whatsoever or as moving physically or being physically moved | ^e^ |  | 0^k^ | 0.0^k^ |  | 0^k^ | 0.0^k^ |

**Notes to S1 Table**

*Note*. - = not applicable. Codes/code combinations that were assigned to the personal experience and/or prototype response of at least 25% of the participants are highlighted in bold.

^a^ *n* = 103 due to missing data (no response to questions on prototype).

^b^ Original responses are in German and were translated/paraphrased by the first author.

^c^ *n* = 97 for personal experience due to missing data (no time specified).

^d^ We coded for involvement (participant in versus witness of event) and psychological distance as defined by Trope, Y., & Liberman, N. (2010). Construal-level theory of psychological distance. *Psychological Review, 117*(2), 440–463. <https://doi.org/10.1037/a0018963>. As we were focusing on involvement, we collapsed codes 322 and 323 as well as codes 332 and 333, because these all code for being in a witness role but with different levels of psychological distance.

^e^ Examples cannot be given because this coding is based on each participant’s entire set of responses.

^f^ The original coding distinguished between the appraisals made by the participant and appraisals that the participant assumed others might make or might have made in this situation. As the latter codes hardly ever occurred, we collapsed the two code categories for each appraisal.

^g^ *n* = 104 for personal experience due to missing data (no bodily symptoms described).

^h^ *n* = 105 for personal experience due to missing data (no cognitive consequences described).

^I^ The original coding distinguished between actions that were intended or deemed to be desirable or appropriate and actions that were actually taken by the participant. We collapsed the two codes to obtain more inclusive code categories.

^j^ *n* = 103 for personal experience due to missing data (no action tendencies described).

^k^ The three participants whose responses received this code were not included in the sample of *N* = 106 analyzed in this paper.
